# Identification of robust deep neural network models of longitudinal clinical measurements

**DOI:** 10.1038/s41746-022-00651-4

**Published:** 2022-07-27

**Authors:** Hamed Javidi, Arshiya Mariam, Gholamreza Khademi, Emily C. Zabor, Ran Zhao, Tomas Radivoyevitch, Daniel M. Rotroff

**Affiliations:** 1 Department of Quantitative Health Sciences, Lerner Research Institute, Cleveland Clinic, Cleveland, OH USA; 2 Department of Electrical Engineering and Computer Science, Cleveland State University, Cleveland, OH USA; 3 Endocrinology and Metabolism Institute, Cleveland Clinic, Cleveland, OH USA; 4 Cleveland Clinic Lerner College of Medicine, Case Western Reserve University, Cleveland, OH USA

**Keywords:** Outcomes research, Type 2 diabetes

## Abstract

Deep learning (DL) from electronic health records holds promise for disease prediction, but systematic methods for learning from simulated longitudinal clinical measurements have yet to be reported. We compared nine DL frameworks using simulated body mass index (BMI), glucose, and systolic blood pressure trajectories, independently isolated shape and magnitude changes, and evaluated model performance across various parameters (e.g., irregularity, missingness). Overall, discrimination based on variation in shape was more challenging than magnitude. Time-series forest-convolutional neural networks (TSF-CNN) and Gramian angular field(GAF)-CNN outperformed other approaches (*P* < 0.05) with overall area-under-the-curve (AUCs) of 0.93 for both models, and 0.92 and 0.89 for variation in magnitude and shape with up to 50% missing data. Furthermore, in a real-world assessment, the TSF-CNN model predicted T2D with AUCs reaching 0.72 using only BMI trajectories. In conclusion, we performed an extensive evaluation of DL approaches and identified robust modeling frameworks for disease prediction based on longitudinal clinical measurements.

## Introduction

Healthcare is undergoing a paradigm shift toward precision medicine treatment decisions tailored to the individual^1^. This shift has been enabled by large volumes of data accruing from a variety of sources including electronic health records (EHRs), genomic sequencing, telemetry, medical imaging, and medical devices. Precision medicine aims to use such data to, for example, identify individuals at risk of developing specific diseases or those likely to respond to a particular treatment. Despite this promise, predictive individualized models remain underutilized in clinical practice^2–4^, as accurate and reliable outcome predictions are rarely achieved. Machine learning has the potential to revolutionize healthcare by transforming large volumes of non-integrated data into knowledge that can aid healthcare provider decision making^5^. When appropriately developed, machine learning models enable sophisticated and objective approaches to high-dimensional multi-modal biomedical data^5^. Currently, there is no gold-standard approach, and models of clinical data are developed and evaluated for specific diseases. Although selecting a specific model architecture should be data- and context-driven, using simulated datasets to compare model performance can provide an objective evaluation of model performance for specific contexts, such as which models are more robust to missingness and changes in trajectory shape. Identifying robust model architectures for specific types of EHR data can help to expedite and improve the development of predictive models.

A critical aspect of precision medicine is being able to predict a disease accurately and precisely in its initial stages^2,3^. To accomplish this, it will be important to leverage all available data. Most models do not take full advantage of longitudinal data available in EHRs, instead using only a snapshot of the most recent data^6–8^. Previous analyses demonstrated how longitudinal information can contribute to risk estimation, e.g., how variability in blood glucose levels can be used to inform risks of microvascular complications in patients with type-2 diabetes^9,10^.

Deep learning (DL)^11^ is a subfield of machine learning that is focused on algorithms inspired by artificial neural networks^12^. In recent years there has been an explosion of DL approaches, including many that utilize longitudinal or time-series data for prediction^13–15^. To compare these methods, it is necessary to evaluate them on simulated data: (1) when real data are used, true membership is unknown (e.g., a false positive may be someone who has not yet been diagnosed). (2) simulated data provide control over numerous statistical parameters (e.g., effect size, class balancing, dispersion, data missingness). This enables assessments of predictor performance dependencies on these parameters. Developing simulated data that adequately represents real data of interest is, however, challenging. Although simulated data have been used to evaluate time series-based DL methods, these simulated datasets were designed for audio and video signal processing^16,17^. EHR data often has correlation structures, non-random missingness, and other characteristics that distinguish it from previously simulated data^18^. Previous studies have simulated clinical event data from EHR data (e.g., diagnosis, medication, procedure codes), but commonly used clinical measurements such as laboratory tests or vitals have not been investigated^19,20^.

Here, for the first time, simulated data based on real body mass index (BMI) trajectories, glucose, and systolic blood pressure (SBP) are used to rate how well DL approaches classify patients based on trajectory changes in magnitude and shape. This enables benchmarking methods across parameters, while maintaining a correlation structure representative of EHR data. We evaluated the performance of nine DL approaches for time-series classification with three main architectures widely adopted for end-to-end DL models:^11^ Multilayer Perceptron (MLP), Convolutional Neural Network (CNN), and Recurrent Neural Network (RNN).

We focused on models that use a supervised approach to training. Here, the model learns by interrogating the mapping between an input time series and the corresponding output to make predictions. These models either have manually engineered features or they are end-to-end models. The former represents each time series as an image^21^ or a forest^22^ that is then used by a DL classifier. The latter identifies the most discriminative representation on its own^23^. In recent years, CNNs, MLPs, and RNNs have all shown promise as predictors of disease risks from EHR^14,24,25^. Our goal in this study was to systematically compare their performances, and the dependencies thereof on EHR data conditions, as such a study has not yet been reported.

We approached this goal in two steps. First, we performed simulations designed to identify DL models that are robust under various conditions. Second, we evaluated the performance of the best performing DL model on real EHR data to predict pediatric type-2 diabetes (T2D). This study advances the state-of-the-art by identifying a DL approach that accurately classifies patient disease risks based on longitudinal clinical measurements from EHR data.

## Results

### Simulated cohorts

**Fig. 1:**
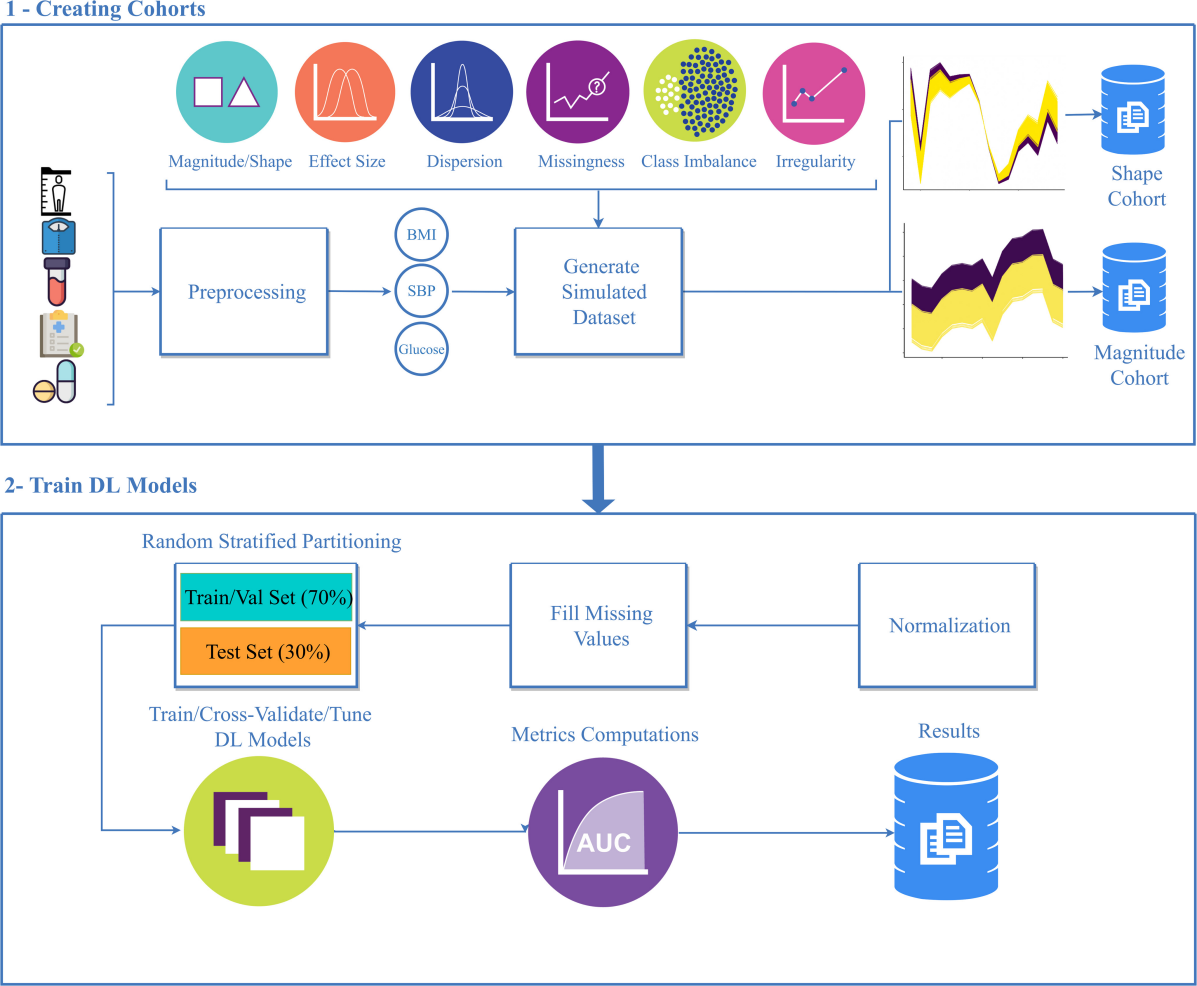
Simulation study workflow. In the first step, longitudinal electronic health record (EHR) data were collected from six randomly selected patients and used to generate reference body mass index (BMI) trajectories using weight and height measurements. Magnitude and shape simulation cohorts were generated from the reference trajectories. Subsequently, simulation cohorts are used to train deep learning models after Z-score normalization, missing value imputation, and partitioning data into training/test sets. Cohorts were randomly partitioned to have training/validation, and test sets of 70%, 30% respectively.

Cohorts were simulated to study predictor performance dependencies on trajectory differences in BMI, glucose, and SBP magnitude and shape (Fig. 1), class distribution overlap, and missingness, irregularity, and class imbalance.

#### Differences in trajectory magnitude

**Fig. 2:**
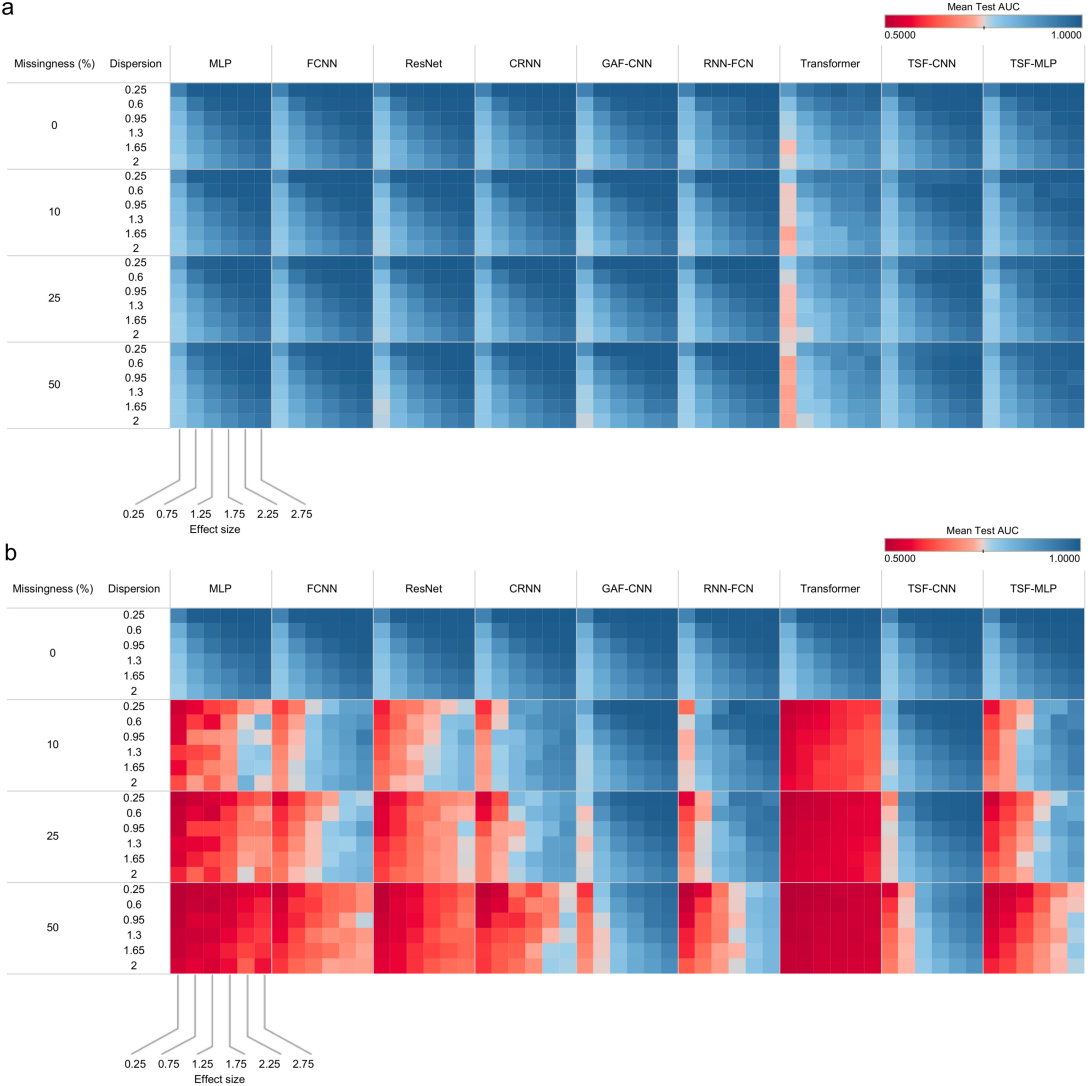
Model predictive accuracies on simulated datasets by effect size, dispersion, and missingness. Cohorts include all class imbalance ratios, with no irregularity present (**a**) Mean area under the receiver operating characteristic curve (AUC) of the test set for the simulated magnitude cohort across BMI, SBP, and glucose cohorts. **b** Mean Test AUC for the simulated shape cohort across BMI, SBP, and glucose cohorts. The x-axis represents different effect sizes, and the y-axis represents different measures of dispersion, categorized by % data missingness. The colors are scaled by the mean AUC for each model.

Models were evaluated based on their ability to classify trajectories due to differences in magnitude across a multitude of effect sizes, dispersions, and fraction of data missingness. Overall, models performed similarly at detecting differences in trajectory magnitudes (Fig. 2a). All DL models, except for the relatively poor performance of the Transformer model, had AUCs ranging from 0.76 to 1.00 across simulations varying in magnitude, minimal differences were observed between models for each magnitude-based simulated scenario, demonstrating comparable performances to each other. Nevertheless, TSF-CNN outperformed other approaches. Notably, despite GAF-CNN still having relatively high accuracies in the magnitude simulation cohort and being the best at detecting differences in shape (described below), it was ranked 8th out of the 9 models in the magnitude cohort (Fig. 2a).

#### Differences in trajectory shape

Substantial variation in model performance was observed when trajectory shapes were modified (Fig. 2b). Except for the GAF-CNN, all other models had at least one AUC = 0.5, meaning performance was equivalent to random assignment. TSF-MLP and Transformer were the worst performers with multiple low AUCs when the missingness >0%, dispersion <1.5, and effect size <1.25 (Fig. 2b). GAF-CNN and TSF-CNN clearly outperformed other approaches. The GAF-CNN demonstrated the best performance with AUCs >0.74 for the most conditions; despite a few low AUCs ranging from 0.6 to 0.7 when missingness = 50%, and effect size = 0.25, where it still outperformed other models. TSF-CNN was second-best with AUCs of ≥0.77 when missingness is <25%; other models had at least one AUC of under 0.7 under the same conditions.

#### Class overlap

Variations in distributional dispersion and effect sizes resulted in a variety of distributional class overlaps for evaluation. Classes with greater class overlap are expected to be more difficult for the model to distinguish because more data points are shared by the two distributions. The AUCs for all models by extent of class overlap with 0% missingness and no irregularity are depicted in Supplementary Fig. 11. In the magnitude simulation cohorts, AUCs of models, except Transformer were ≥0.94 when class overlapping is <90%. Transformer had AUCs of >0.88 for the same condition. AUCs decreased from 0.95 to 0.86 (0.82 for Transformer) for magnitude cohorts that overlapped between 90 and 100%.

For the shape cohorts, AUCs of models ranged from 0.85 to 1 for 100–0% distribution overlap. All models had AUCs of >0.99 for overlaps of <40%, and AUC of >0.94 for overlaps of <90%.

#### Data missingness

**Fig. 3:**
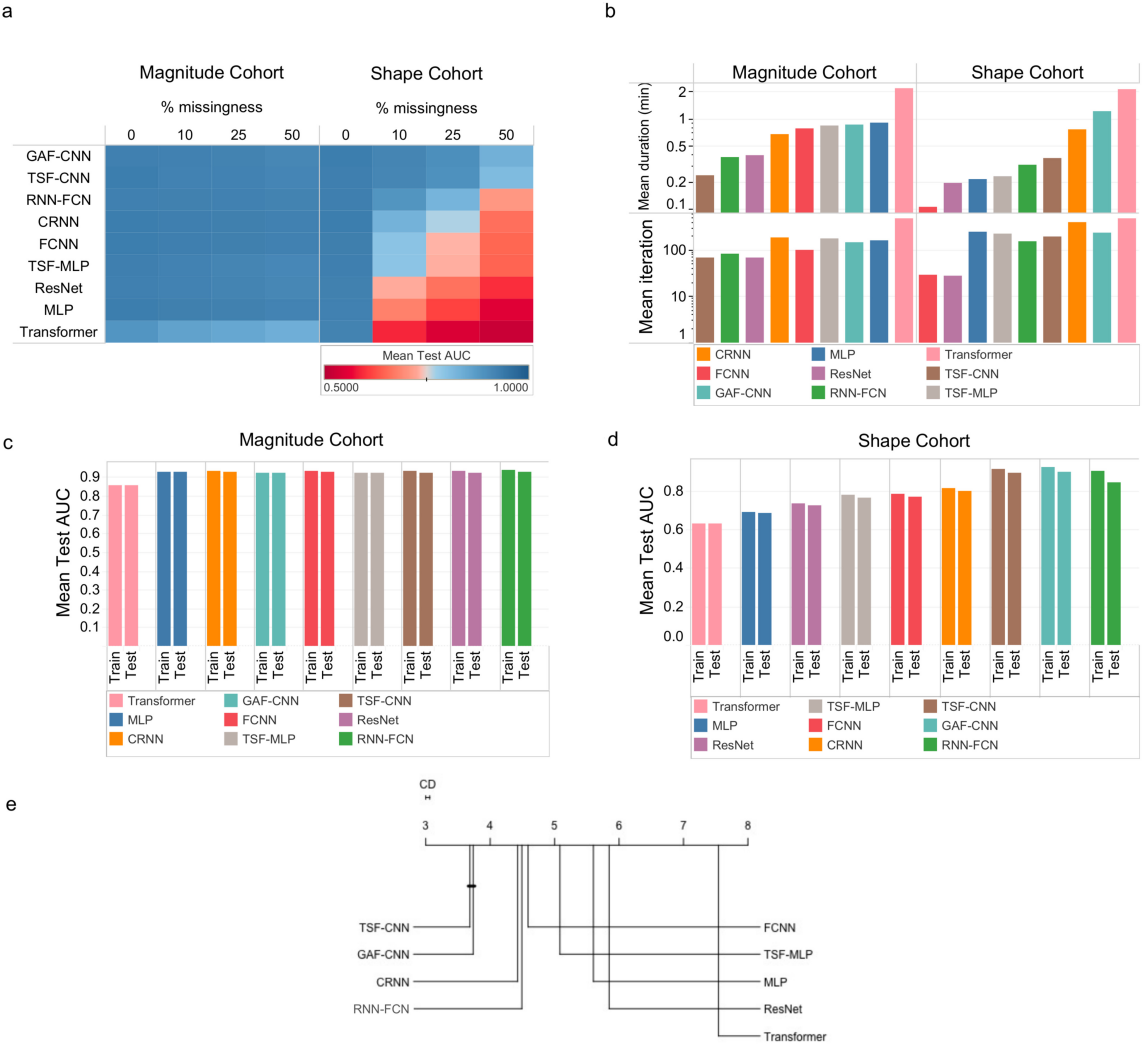
Model performance on simulated data by overfitting and training time. **a** Area under the receiver operating characteristic curve (AUC) differences of train and test sets over different levels of missingness. **b** Training durations and iterations. **c** Model overfitting was evaluated by comparing the AUC differences for each deep learning method in the training sets and the test sets based on (**c**) changes in trajectory magnitude and (**d**) changes in trajectory shape. **e** Critical difference plot to compare model’s performance based on their test AUC (Lower CD value is better).

We investigated the impacts of data missingness on model prediction accuracies based on missingness of 0, 10, 25, and 50%. In the magnitude simulation cohort, all models, with the exception of Transformer that had AUCs from 0.84 to 0.88, demonstrated similar AUCs of ~0.93 for all four categories of missingness (Fig. 3a).

In the shape simulation cohort, GAF-CNN was robust over various degrees of missingness with AUCs of 0.93, 0.92, 0.90, 0.83 for missingness of 0%, 10%, 25%, and 50%, respectively. TSF-CNN performed comparably with AUCs ranging from 0.93 0.91, 0.89, and 0.81 for 0%, 10%, 25%, and 50% missingness (Fig. 3a). Models that were the most detrimentally impacted by missingness were Transformer, MLP, and ResNet with respective AUCs of 0.52, 0.55, and 0.59 at 50% missingness

#### Data irregularity

We investigated the impacts of data irregularity (i.e., irregular spacing between patient measurements) on model prediction accuracies. In the magnitude simulation cohort, with the exception of the transformer model, models demonstrated similar accuracies across irregularity classifications, with AUCs ranging from 0.84 to 1.00 (Supplementary Fig. 12).

In the shape simulation cohort, GAF-CNN was robust over various degrees of irregularity with lowest AUCs of 0.85, 0.82, and 0.82 for no, moderate, and high irregularity, respectively (Supplementary Fig. 12). TSF-CNN demonstrated similar performance with its lowest AUCs of 0.85, 0.81, and 0.80 for no, moderate, and high irregularity, respectively. The RNN-FCN model also performed quite well in this regard with its lowest AUCs of 0.85, 0.79, 0.76 for no, moderate, and high irregularity, respectively. Models that were the most detrimentally impacted by irregularity were TSF-MLP, MLP, FCNN, CRNN, ResNet, and Transformer with respective lowest AUCs of 0.59, 0.58, 0.65, 0.68, 0.62, 0.56 at high irregularity.

#### Class imbalance

Class proportions in EHR data are often imbalanced, with fewer people having a record of the outcome of interest. In our simulation dataset, we varied the class imbalance class ratio from 1 to 0.25 representing the minority class. As the imbalance ratio decreases (i.e., minority class is smaller relative to the majority class), worse model performance is expected due to the need to downsample the majority class to have balanced model training. The AUCs for all models by extent of class imbalance is depicted in Supplementary Fig. 13. In the magnitude simulation cohorts, AUCs were ≥0.92 across all models and for all four scenarios of class imbalance.

For the shape cohorts the GAF-CNN model demonstrated the highest AUCs, with AUCs of 0.91, 0.91, 0.90, and 0.88 for 1, 0.75, 0.5, and 0.25 class imbalance ratios, respectively. TSF-CNN was the second highest performing model with AUCs of 0.91, 0.90, 0.89, and 0.86, respectively (Supplementary Fig. 13).

#### Overall accuracy

Overall model accuracy was determined using a Friedman test to evaluate model AUCs across all simulated conditions and is depicted as a critical difference plot (Fig. 3e). TSF-CNN was ranked as the best model but was not statistically significantly better than GAF-CNN, which was ranked second (*P* > 0.05). These models were followed by CRNN, LSTM-FNC, FCNN, TSF-MLP, MLP, ResNet, and Transformer, respectively, and these were all statistically significantly different from each other (*P* < 0.05) (Fig. 3e).

#### Model training time

We compared models based on average training duration and average training iterations, stratified by magnitude and shape cohorts (Fig. 3b). In the magnitude simulation cohort, the TSF-CNN model learned fastest with a mean duration of 0.24 min (14.4 s) for each individual cohort within the dataset. Other models except Transformer were also fast learners with mean durations of <1 min. The longest training time was observed with the Transformer which had a mean duration of 2.17 min. In the shape simulation cohort, the fastest model was the FCNN with a mean duration of 0.1 min (6 s). MLP, TSF-MLP, ResNet, TSF-CNN, and RNN-FCN were also fast learners with mean durations <0.37 min (22.2 s). Consistent with the magnitude cohorts, the model with the longest training time was the Transformer with a mean duration of 2.16 min (Fig. 3b).

TSF-CNN, RNN-FCN, ResNet, and FCNN had <100 iterations (i.e., epochs) in the magnitude cohort to meet the stopping criteria (Fig. 3b). The maximum number of iterations was observed with the Transformer, which had a mean of 500 iterations. In the shape cohorts, FCNN and ResNet met the stopping criteria in <29 iterations (Fig. 3b). Other models met stopping criteria later but with <400 iterations. Similar to the magnitude cohorts, the Transformer did not meet the stopping criteria and reached the maximum allowed 500 iterations.

#### Model overfitting

We calculated the difference in model AUCs obtained in the test set and compared them to the training set for each simulated cohort to determine the extent of model overfitting. All DL models demonstrated similar accuracies between the training and test sets in the magnitude cohorts (AUCs ~0.92). The difference in AUCs between the test and training sets were negligible with the highest difference observed with ResNet, which had a mean AUC difference of 0.0015 (Fig. 3c). More substantial model overfitting was observed in the shape cohort. TSF-CNN demonstrated the least model overfitting with a difference in AUC of 0.0051, and RNN-FCN demonstrated the greatest model overfitting with a difference in AUCs between training and test cohorts of 0.0227 (Fig. 3d).

### Real-world, pediatric type-2 diabetes prediction based on BMI

**Fig. 4:**
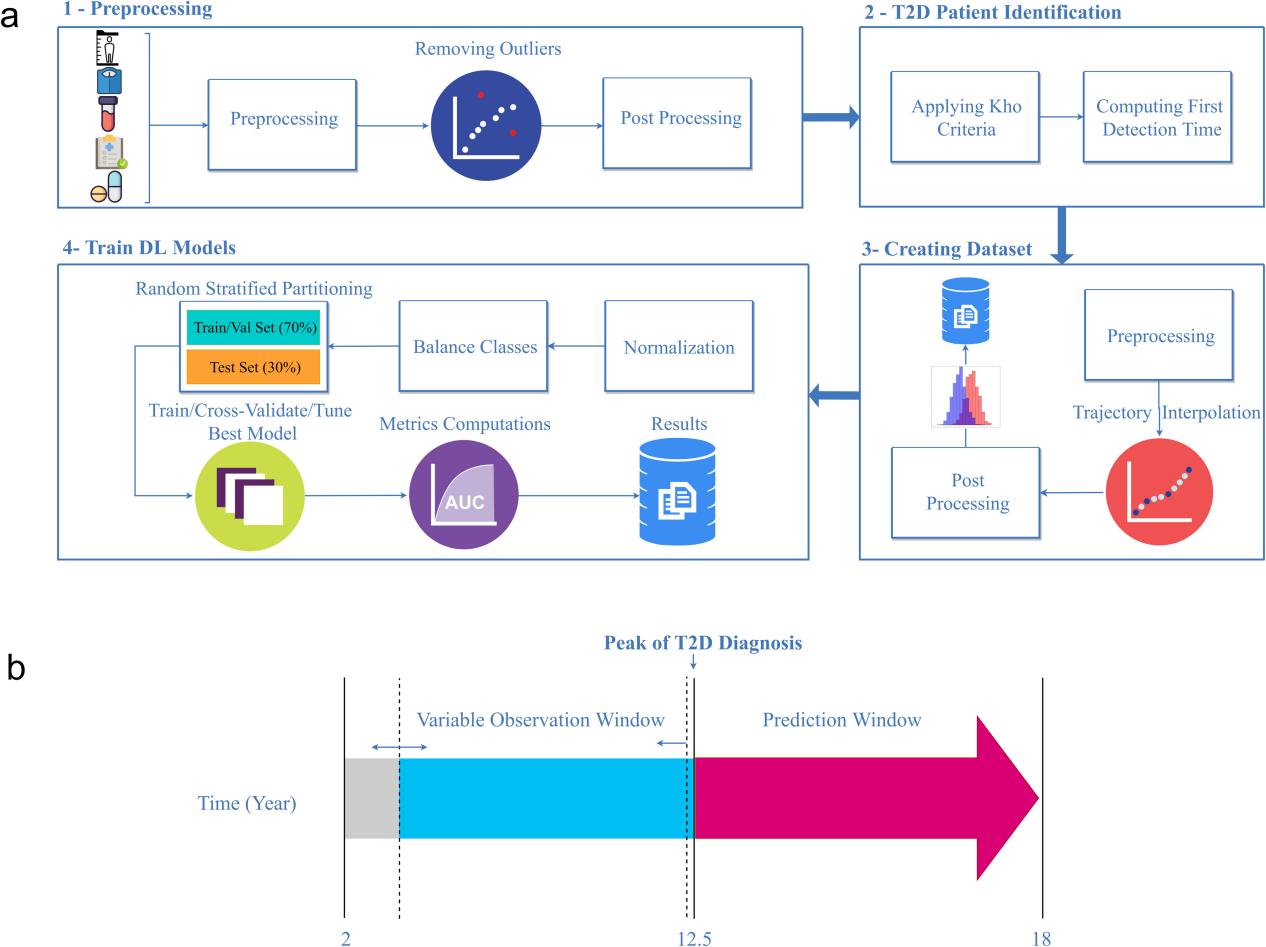
Modeling real-world pediatric type-2 diabetes (T2D) workflow. **a** Longitudinal weight and height data were extracted from the EHR. Height and weight outliers were removed and patients with <2 BMI records were excluded. Next, patients were labeled as underweight, normal weight, overweight, obese, and severe-obese according to Centers for Disease Control criteria, and were classified as those that developed T2D or did not develop T2D according to the eMERGE criteria^44^. Trajectories were then harmonized such that each trajectory consisted of a single BMI record per year. Trajectories were truncated to exclude any records at or after the average age of pediatric T2D onset and any records after a patient’s T2D diagnosis. Because deep learning models require uniform trajectory lengths, multiple datasets were created across a variety of different age ranges. For each dataset a TSF-CNN model was tuned using 20% of the training set and trained using tenfold cross-validation; the mean and standard deviation of validation and test AUCs were calculated. Subsequent modeling steps were consistent with those shown in Step 2 of Fig. 1. **b** Depicts the observation and prediction window definitions. The observation window varied from 2 years old to average age of T2D in pediatric patients (12.5 years old), and the prediction window was fixed from 13 to 18 years old.

A total of 35,056 pediatric patients met our inclusion/exclusion criteria based on their available EHR data (Fig. 4). A detailed STROBE diagram showing the number of patients included/excluded at each processing stage is shown in Supplementary Fig. 9. The final cohort was 45.8% female, with an average age of 5.4 years old at the first clinical record and an average age of 17.1 years old at the last clinical record. The average length of BMI trajectories spanned 8.2 years. Overall, 41.3% and 24.7% of the cohort met the criterion for obesity and overweight, respectively, as defined by Centers for Disease Control 2000 growth curves^26^. The TSF-CNN outperformed other evaluated models in the simulated cohorts, and for this reason, was used to predict pediatric T2D. The detailed preprocessing steps are shown in Supplementary Fig. 10.

**Fig. 5:**
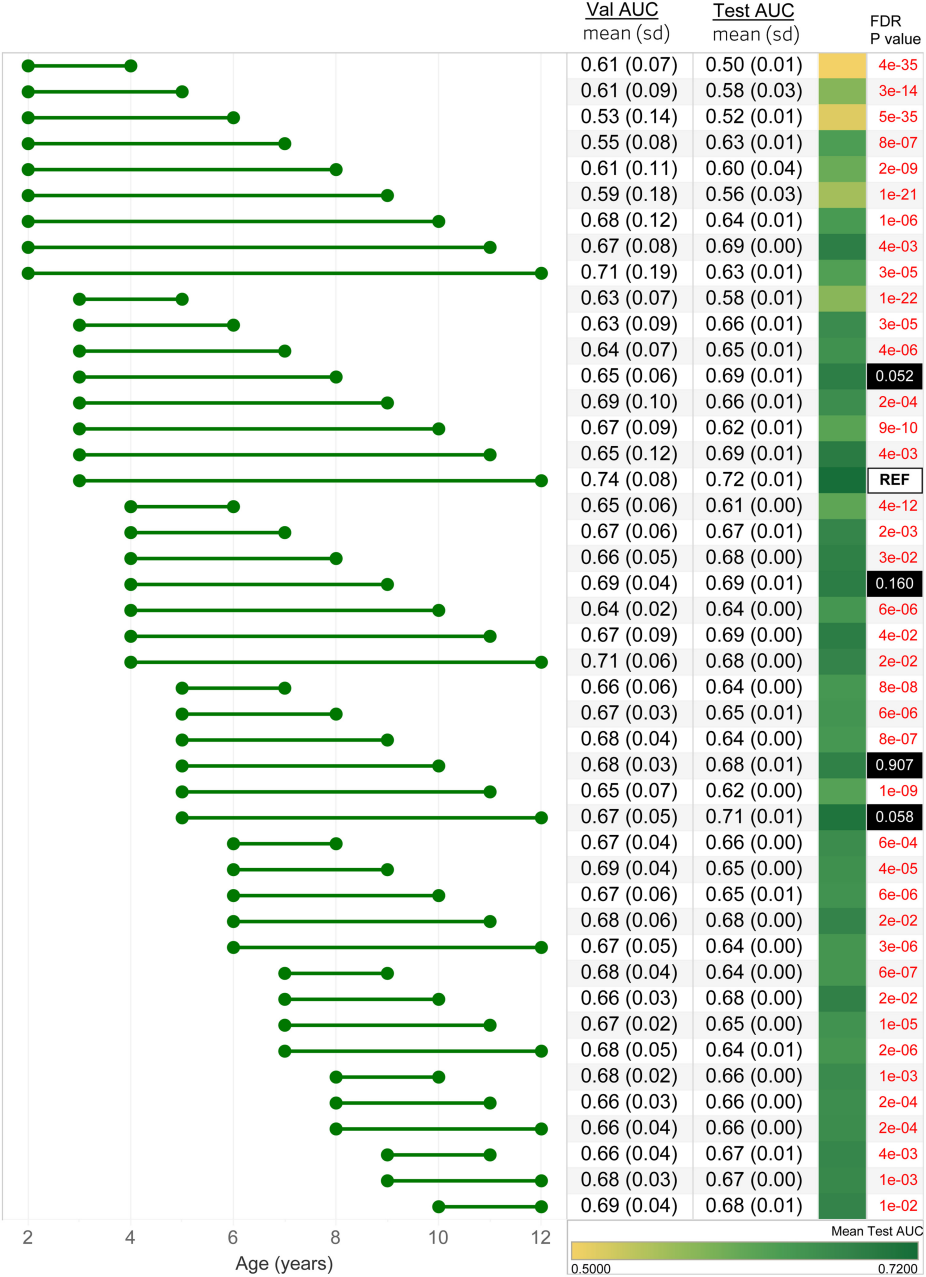
Performance of the TSF-CNN model to predict pediatric type-2 diabetes (T2D) in a real-world cohort. Different age ranges incorporated in the model are shown with the horizontal bars. Mean and standard deviation of cross-validation (Val) AUCs and test AUCs are shown. The third column represents the model area under the receiver operating characteristic curve (AUC when it is applied to the withheld test set) with a green gradient indicating the accuracy of the prediction. The fourth column indicates the FDR *P* values of the Delong’s test for each AUC compared to the best performing age range, ages 3–12, annotated with “REF”. Models with a FDR *P* < 0.05 are shown in red.

A total of 2336 out of 35,056 patients (6.6%) met the criteria for T2D and were labeled as positive cases. We investigated 51 different observation windows for predicting T2D depending on the extent of patient data available (Supplementary Table 1). In general, model performance improved when the observation window included a wider range of ages, and the max age was closer to the maximum of 12 years old (Fig. 5). The model with the observation windows spanning 3–12 years old had the highest accuracy (AUC = 0.72). However, models constructed on ages 5–12 (AUC = 0.71), 3–8 (AUC = 0.69), 4–9 (AUC = 0.69), 5–10 (AUC = 0.68) were not statistically different from the best performing model (FDR *P* < 0.05) (Fig. 5). Models incorporating fewer and younger ages performed worse with the observation window spanning 2-4 demonstrating the lowest accuracy (AUC = 0.50) (Fig. 5).

## Discussion

EHRs are contributing to an explosive growth in diverse longitudinal clinical data that will increasingly support population-level analyses to extract similar patterns among patient records for disease prediction. Predictive analytics represent an important domain with goals that include early detection of diseases, avoidable care, and clinical decision support. The performance of a predictive model, represented by the AUC for categorical endpoints, is a dominant focus, but there are other factors (e.g., overfitting, robustness to missingness, time to train) that will dictate the overall utility of a model and thus whether it will be adopted for clinical care. Although many systematic comparisons of DL approaches using longitudinal data have been presented^27–31^, to our knowledge, this is the first study specifically evaluating DL models using longitudinal clinical data of lab measurements or vital data. Patient clinical histories have different correlation structures and specific challenges (e.g., missing data) that may be best met by models that are different than those applied to audio or video signaling data and other general forms of time-series data that are typically used in model comparisons^27–29^. Simulation experiments where the true signal is known are critical to evaluating method performance. Here we took a semi-synthetic approach where the signal and noise in the data were simulated, but the correlation structure was maintained^32^. This approach enables interrogation of method performance, while maintaining a simulated cohort that resembles what may be observed clinically.

Based on the results of this study, we can provide guidance on what DL approaches are best suited for predictions from longitudinal EHR data from common clinical measurements, such as clinical labs and vitals. If the goals are to predict based on magnitude changes in trajectories, all models performed comparably well with >0.76 AUC across simulated cohorts (Fig. 2a), with the TSF-CNN approach learning most efficiently (Fig. 3b). Although, GAF-CNN still had relatively high AUCs for detecting changes in magnitude, it ranked 8th out of the 9 models in this category (Fig. 2a). As the overlap, irregularity, and class imbalance ratio increase, discriminating the correct class for an input trajectory becomes more difficult. For all models, discriminating classes from distributions that overlapped by >90% was comparably difficult with AUCs dropping to 0.82 with no missing data or data irregularity (Supplementary Fig. 11). If differences in trajectory shape are thought to be important predictive features and data missingness is present, GAF-CNN stood out with the highest prediction accuracies when significant amounts of data were missing, with AUCs of 0.90 and 0.83 with 25% and 50% missingness, respectively (Figs. 2b, 3a). Although the GAF-CNN approach performed best for the shape simulation, it was followed closely by TSF-CNN, which were statistically equivalent based on their AUCs across all simulated scenarios (*P* > 0.05) (Fig. 3e). The TSF transformation uses summary statistics of various trajectory intervals (e.g., mean, standard deviation, slope) and appears to be particularly well-suited for capturing information from clinical measurement trajectories to form predictions (Supplementary Fig. 4).

The number of training iterations required to reach an optimal prediction accuracy provides insight into how efficiently a model learns. This is important as EHRs can contain millions of patient trajectories, and in these circumstances, efficient learning is critical. Although the iterations were similar for the other models, Transformer and GAF-CNN have the deepest and most complex architecture, which required longer training durations. Although GAF-CNN showed competitive performance to TSF-CNN, it suffered from time complexity, which becomes a significant limiting factor for large cohorts. FCNN was overall the most efficient learner across all simulation cohorts, followed closely by ResNet RNN-FCN, and TSF-CNN (Fig. 3b).

Although the simulation cohorts utilized for this study were designed to interrogate common clinical measurement data (*i.e*., BMI, SBP, glucose), across various effect sizes, missingness, dispersion, irregularity, and class imbalances, there were limitations that present opportunities for future studies. We only investigated a two-class problem, and although two classes (disease/no-disease) are typical for clinical prediction models, there are other scenarios that could benefit from multi-class prediction or prediction of continuous outcomes. In addition, the data used in this study originated from a single, large integrated health system. Future evaluations may benefit from using data across multiple health systems to ensure the results are broadly applicable. Although the names for many of the DL architectures have remained consistent, the network architectures themselves are evolving. As examples, MLP (Multilayer Perception) and VGG (a CNN architecture) have recently evolved in ways that improve their performance^33,34^. Therefore, the conclusions to be drawn from the current study may evolve over time.

All models evaluated here require complete data (i.e., no missing values) and uniform trajectory lengths. This is a challenge because patients have clinical histories of different lengths and have visits at different intervals. We addressed the missing data limitation directly as part of our simulation strategy, and approached the limitation of different lengths of trajectories in the real-world data by training multiple models on different patient observation windows (Fig. 4b, Supplementary Fig. 12). The advantage of this approach is that predictive accuracy can be evaluated based on the extent of data available for a patient, but the disadvantage is that multiple models need to be developed to accommodate a wide range of patients in an EHR. Notably, the TSF-CNN model was able to predict risk of pediatric T2D based on BMI alone with mean test AUCs of up to 0.72 using BMIs from 3 to 12 years old (Fig. 5). As expected, models incorporating BMIs at later age ranges, and with larger cohort sizes, demonstrated higher predictive accuracies. In this case, some of the longest trajectories did not perform as well due to fewer patients having medical histories spanning that many years. However, when models trained on data when the prevalence of cases were ≥5%, trajectories were more accurate when earlier data points were included. For example, BMIs from 5 to 12 years old predicted T2D with AUC = 0.72, whereas 10–12 years old predicted T2D with 0.68 (FDR *P* < 0.05). Previous studies showed that high childhood BMIs are associated with adult type-2 diabetes and metabolic syndrome^35,36^. However, there are no systematically used prediction models for pediatric T2D using EHR data. Additional work is needed to leverage this approach, along with additional clinical history and laboratory data, to optimize childhood T2D predictions in clinical settings.

Here, we explored nine DL approaches to identify the most robust approach for constructing predictive models from longitudinal clinical measurements from EHRs, such as BMI, glucose, SBP. We evaluated performance dependencies on trajectory shape, trajectory magnitude, effect size, dispersion, class overlap, irregularity, class imbalance, and data missingness. From these results, we identified TSF-CNN and GAF-CNN as promising frameworks for using longitudinal clinical measurements in predictions and illustrated this using longitudinal BMI trajectories from pediatric patients to predict T2D onset.

## Methods

### Simulated cohorts

To ensure the data accurately represented real-world data, we simulated BMI, glucose, and SBP trajectories based on six real patient trajectories for each group to ensure relevant correlation structures. The trajectories were randomly selected from a pool of patients in the Cleveland Clinic EHR seen between 2000 and 2018. If the selected trajectory was missing a yearly measurement, the missing point was imputed with the mean of the immediately preceding and succeeding values. Each trajectory was then median normalized. We created separate cohorts to evaluate DL classifications based on trajectory shapes or trajectory magnitudes. Simulated magnitude cohorts were derived by random sampling from a normal distribution of magnitudes of a corresponding mean (effect size) and standard deviation (dispersion). Simulated shape cohorts were created by fitting a polynomial regression model with ten coefficients on the patient trajectory using *poly* function in R^37^. We then determined the model coefficient with the greatest influence on trajectory by permutating each model parameter 1000 times and calculating the mean squared error (MSE) between the new shapes and the original shape. The coefficient that resulted in the greatest average MSE upon permutation was considered the most influential for modifying the trajectory shape. Simulated shape cohorts were then created by modulating this influential parameter by random sampling from a normal distribution of coefficients of a corresponding mean (effect size) and standard deviation (dispersion). Simulation across each set of conditions resulted in 6336 magnitude cohorts and 6336 shape cohorts representing variations in effect size, dispersion, class overlap, irregularity, class imbalance, and missingness. Each cohort consisted of two classes of up to 2000 trajectories in each class with a maximum length of 16 data points. Effect sizes were modulated by differences in the means of the distributions (0.25–2.75, step size = 0.5). The dispersion was modulated by changing the standard deviations of the distributions (0.25–2, step size = 0.35). Missingness was simulated by randomly dropping varying numbers of data points along each trajectory (i.e., 0, 10, 25, 50%). The distributional overlap between the two classes was calculated as the proportion of trajectories that had values common to both classes at all time points. Simulated missing values were then imputed using the commonly utilized approach, last observation carried forward (LOCF). If the initial observation was missing, then the last observation carried backward (LOCB) was used. We investigated the impact of data irregularity (i.e., random irregular spacing between patient measurements) on model prediction accuracy. A beta distribution was used to sample the number of missing data points applied to each individual’s trajectory. Irregularity was classified as no irregularity, meaning all patient measurements were equally spaced, moderate irregularity, meaning patients had between 0 and 3 missing measurements (mean = 1) or high irregularity, meaning patients had between 2 and 5 missing measurements (mean = 4). The distribution of missing data points for patients in the moderate and high groups, along with specific beta distribution parameters, are provided in Supplementary Fig. 14. Moreover, we analyzed the impact of class imbalance on model prediction accuracies. We randomly down-sampled individuals from one class, while keeping individuals from the other class the same, to create four levels of class imbalance—0, 10, 25, and 50%. In this case, 0% is equally balanced and 50% means the size of one class is half the other class.

As part of standard data processing, the data were Z-score normalized prior to model training except for the GAF-CNN model, which were min–max normalized. Cohorts were then randomly partitioned into training/validation (70%), and test sets (30%), respectively. We randomly selected 20% of the training set to perform each model’s hyperparameter tuning. A workflow depicting the process for developing the simulated cohorts is presented in Fig. 1. To compare the performance of models, we consider two performance metrics: 1—area under receiver operating characteristic curve (AUC) showing how well a model can distinguish between classes and 2—training duration calculated by the product of the training time per epoch and the number of epochs needed to reach the desired level of accuracy. System specifications used to train the models are provided below. Additional metrics such as the F1-score (i.e., dice coefficient), precision, recall (sensitivity), and specificity are available in Supplementary File 1.

### Deep neural network models

#### Multilayer perceptron (MLP)

We implemented a simple and traditional MLP architecture, proposed in Wang et al. as the baseline approach for time-series classification (Supplementary Fig. 1). This model consisted of four layers, and was optimized using the Adadelta method^27^. The final layer has two neurons to accommodate a binary classifier with a softmax activation function. The three hidden fully connected layers consisted of 500 neurons with a Rectified Linear Unit (ReLU) activation function. To reduce overfitting, prior to each of the three hidden layers we included dropout layers with respective rates of 0.1, 0.2, and 0.3. An important aspect of the MLP is that the number of neurons does not depend on time-series length; total weights do, however, depend on time-series length.

#### Fully convolutional neural network (FCNN)

FCNNs have been proposed for classifying univariate time series^27^. A CNN model is usually composed of multiple convolutional layers followed by a few fully connected layers. FCNN is a CNN without local pooling layers, which maintains the length of the time series after each convolution. In addition, the final fully connected layer is replaced with a global average pooling (GAP) layer, which significantly decreases the number of parameters. We implemented the architecture of Wang et al.^27^ which has three convolutional blocks with a ReLU activation function, and 128, 256, and 128 filters with filter lengths eight, five, and three, respectively (Supplementary Fig. 2). The GAP layer averages the results of the last convolution layer over the entire time dimension and feeds it into the softmax classifier in the fully connected output layer. A major advantage of the FCNN approach is that the number of parameters for four layers is invariant to time-series length due to the inclusion of a GAP layer in the network.

#### Time series forest MLP (TSF-MLP)

The time-series forest (TSF) algorithm extracts statistical features from random periods of each time series to engineer features prior to model training^22^. TSF divides the time series into small contiguous intervals that have a certain length and a random start position. From these intervals, the mean, standard deviation, and slope of the random intervals are calculated and used as model features^22^. An advantage of this algorithm is that TSF is robust to time-series noise because it uses a phase dependent discriminatory subseries instead of the whole series, reducing the potential bias from noise in the trajectory^29^. We designed a classifier which leverages a MLP classifier and the TSF algorithm (Supplementary Fig. 3). TSF outputs were used as features for a MLP model with the architecture described above. Therefore, instead of training the model directly on the time-series data, the model was trained on engineered features from the TSF algorithm.

#### Time series forest CNN (TSF-CNN)

Similar to the TSF-MLP described above, we leveraged features engineered by the TSF algorithm and implemented a CNN with six one dimensional convolution layers with 32, 32, 32, 32, 64, 128 filters, each with filter lengths of three with the ReLU activation function. A max pooling layer with stride of two was implemented after the second and fourth layers, which takes the maximum from each set of two values of the input. There is a GAP layer that takes the results of the sixth layer and performs averaging over the entire time dimension, feeding to a softmax classifier in the fully connected output layer (Supplementary Fig. 4).

#### Gramian angular field CNN (GAF-CNN)

Gramian angular field (GAF) is a feature engineering approach. Encoding the time-series value as the angular cosine and the time stamp as the radius transforms the time series into images^21^. The GAFs contain temporal correlations since time increases as the position moves from top-left to bottom-right in the GAF matrix. The main diagonal contains the original information needed to reconstruct the time series from the high-level features learned by the deep neural network. We built a hybrid CNN classifier that takes a GAF of the input time series and has six 2D convolution layers with 32, 32, 32, 32, 64, and 128 filters with dimensions of 3 × 3. Also, there are two down sampling layers and two max pooling layers with stride two after the 2nd and 4th layers. Finally, a GAP layer is utilized before the last layer which is a softmax classifier (Supplementary Fig. 5).

#### Residual network (ResNet)

ResNets capture linear and non-linear dependencies of time-series elements on both short- and long-term scales. ResNet features the shortcut residual connection between consecutive convolution layers which enables the flow of the gradient directly through these connections with the aim of reducing the vanishing effect^38^. The architecture (proposed in Wang et al.^27^) is relatively deep with 3 residual blocks with 3 convolution kernels whose output is added to the residual block’s input and then fed into the next layer. These three blocks are followed by a GAP layer and a softmax layer with two neurons. The length of filters in each residual block is set to 8, 5, and 3 for the first, second and third convolution, respectively. The number of parameters in ResNet is also invariant across different time-series lengths (Supplementary Fig. 6). Although not done here, these features enable transfer learning for the ResNet model, which means it is possible to fine tune a pre-trained ResNet model for an unforeseen dataset.

#### Recurrent neural network—fully convolutional network (RNN-FCN)

RNNs are a DL model with complex architectures designed to predict an output for time stamp in a time series using a gradient based method^39^. A RNN model consists of a chain of modules in a row called long short-term memory (LSTM). RNNs keep the history of all the past time-series elements and use this information as they process an input sequence one element at a time. A limitation of RNNs is that their high computational costs, which may pose a significant challenge for certain applications. We implemented a state-of-the-art LSTM architecture^40^ that is augmented by a fully convolutional block followed by a dropout block. In this architecture, a RNN and a CNN process the input series in parallel and their outputs are joined using a concatenation layer (Supplementary Fig. 6). The fully convolutional block consists of three stacked temporal convolutional blocks with 128, 256, and 128 filters, and filter sizes of 8, 5, and 3, respectively. Each block consists of a temporal convolutional layer, which is accompanied by batch normalization followed by a ReLU activation function. Finally, global average pooling is applied following the final convolution block. The output layer consisted of a dense layer with two neurons with a softmax activation function (Supplementary Fig. 6).

#### Convolutional-recurrent neural network (C-RNN)

We developed a hybrid network structure composed of both a RNN and a CNN to extract comprehensive multifaceted patient information patterns. We designed the hybrid C-RNN by taking advantage of the CNN for extracting spatial information and utilized the RNN structure for its ability to learn temporal relevance in time-series data, as in Wang et al.^41^. This differs from the RNN-FCN approach, which includes an LSTM layer that accepts raw time-series features. Here, the LSTM layer accepts convolved features that may improve its ability to track dependencies in time-series data. The CNN structure included a 32-filter layer, followed by a 64-filter layer, with filter lengths of 5 and 3, respectively. The output feature map is then fed into a RNN structure, which consists of a LSTM block with 64 units and two dropout layers set to 0.1 and 0.5, respectively. RNN outputs are then fed into a softmax classification layer (Supplementary Fig. 7).

#### Transformer

Transformer is a DL model based on an attention mechanism that was originally proposed for machine translation^42^. It is a method that draws global dependencies between input and output by relying entirely on self-attention to compute representations of its input and output without using sequence-aligned RNNs or convolution. We implemented the base model of transformer architecture and applied it to time-series instead of natural language. The model consists of an encoder composed of a stack of 4 identical layers (*N* = 4). Each layer consists of two sub-layers. The first is a multi-head attention and the second is a feed-forward network. A residual connection is attached to each sub-layers followed by layer normalization and a dropout to reduce the output tensor size. The number of attention heads, head size, dropout amount, and the number of neurons of feed-forward are determined in the hyperparameter tuning process (Supplementary Fig. 8).

### Hyperparameter tuning and model training

Cohorts were then randomly partitioned into training/validation and test sets, representing 70% and 30%, respectively (Figs. 1, 2). We randomly selected 20% of the training set to perform each model’s hyperparameter tuning. We performed 3-fold cross-validation for simulated cohorts and tenfold cross-validation for the real-world cohort. The mean and standard deviations of the model training/validation AUCs for the simulated cohorts can be found in Supplementary File 1 and for the real-world cohorts in Fig. 5.

For all DL architectures presented here, we performed hyperparameter tuning that considered multiple dimensions, including dropout and batch size as general parameters, kernel size, kernel width, and number of neurons in each layer for CNNs, dense size for MLPs, LSTM size for RNNs, and the number of heads, head size and learning rate for the Transformer. The bandit-based approach^43^ was used to search through the hyperparameter space to tune parameters during up to 100 iterations (Supplementary Table 3).

We initialized the learning rate number of epochs with respective values of 0.001, and 500. All models were optimized using the Adam method with binary cross-entropy as their cost function. The detailed architectures for all the described approaches are available in Supplementary Table 2. For all models, the learning rate was reduced by an arbitrary factor of 0.92 each time the model’s training loss was unimproved for three consecutive epochs. Although the learning process for each classifier was permitted to take up to 500 iterations, a stopping criterion was implemented across all models; training was stopped if no improvement in model accuracy was gained after 15 consecutive iterations. The model with the best performance on the validation dataset was chosen for evaluation on the test set. The statistical significance of each model’s performance was evaluated using a critical difference plot and a non-parametric, Friedman test across the AUCs from all simulated conditions in the test sets. Models with a *P* < 0.05 were considered to have performances that were statistically significantly different from one another.

### System specification

All models were generated using the Cleveland Clinic’s High Performance Computing Cluster (HPC). We issued multiple jobs which each one includes process of training, tuning, validation, test of a DL model. For each job, we allocated 48 cores consisting of Intel(R) Xeon(R) Platinum 8168 CPUs @ 3.90 GHz 64 bits, with dedicated memory allocation of 128 GB RAM with CentOS Linux v8 operating system.

### Real-world cohort

The model that demonstrated the best performance across the simulated cohorts was evaluated in a real-world pediatric (ages 2–18 years) cohort to predict onsets of pediatric type-2 diabetes, prediabetes, and metabolic syndrome (Fig. 4a). The cohort consisted of 51,164 pediatric patients (54.9% male and 45.1% female based on self-report in the EHR) seen at the Cleveland Clinic between 1987 and 2020. Structured data, including height, weight, demographic information, encounter diagnosis international classification of disease (ICD) 9/10 codes, and medication records were extracted from the EHR. Patients were considered to have T2D based on the visit at which patients met the eMERGE criteria^44^. Patients without a recorded encounter diagnosis or weight and height measurements were excluded. BMI was calculated from height and weight measurements from ages 2 to 12 years old or to the date of pediatric T2D diagnosis, whichever came first. Twelve years of age was selected as the max for BMI records because 12.5 years was the average age of a pediatric T2D diagnoses in this cohort; data prior to the age that most pediatric patients are diagnosed with T2D is desirable so that all models will be broadly applicable to most patients. Patients must also have had at least one encounter ICD code at or after the age of 18 to ensure they were still being seen by providers in the Cleveland Clinic health system. Additional selection criteria were implemented based on data availability and are described in the Data Processing and Quality Control section below. This study was approved by Cleveland Clinic Institutional Review Board (IRB #20-1035) and complied with current ethical regulations, consent was waived due to the retrospective nature of the study. The workflow for cohort selection and data processing is depicted in Fig. 4.

#### Data processing and quality control

All data processing was performed using R 4.1.1^37^ and Python 3.7^45^. Quality control was performed at both the cohort and the individual level. Individuals were excluded if they were outside of the modified Z-score range as described by the Center for Disease Control^46^. According to these criteria, patients with either a body weight Z-score <5 or >8, a height Z- score <5 or >4, or BMI Z-score ≤4 or >8, were excluded. Implausible records, such as height of *≤*25 inches or *≥*100 inches and weight of *≤*5 or ≥1000 lbs. were excluded, as previously described by Boone-Heinonen et al.^47^. Before calculating BMI trajectories, weight and height trajectories were smoothed at the individual level using local polynomial regression with an automatically selected smoothing parameter, as in Tao et al.^48^. Because all the evaluated model architectures require a uniform matrix (i.e., same number of longitudinal data points across all patients), we partitioned the BMI trajectories into disjoint 1-year segments and each segment was assigned a BMI based on the mean of all BMIs recorded within the segment. In addition, we created multiple “observation windows” for different age ranges for which BMI values would be used for prediction, which is needed since patients have varying lengths of medical histories. Having models for multiple observation windows provides performance metrics for prediction accuracies depending on the amount of data available (Fig. 4b). Linear interpolation was used to impute missing records between the first record and the last available record in the trajectory. Patients without a BMI trajectory covering the observation window were excluded.

#### Predictive model development

Multiple TSF-CNN models, selected based on performance in the simulated cohorts, were developed on multiple age ranges (i.e., observation windows) of trajectories (Figs. 4B, 5). This was done to evaluate model performance for different lengths of medical history. No observation windows included BMI values from patient records >12.5 years old (the mean age of pediatric T2D onset in this cohort). Cohorts were partitioned into training/validation (70%), and test (30%) sets. Due to the different age ranges for different models, each model was based on a different cohort size (Supplementary Table 4). TSF-CNN models were evaluated using tenfold cross-validation for each cohort to evaluate the performance accuracy of the model in its stable state. The rest of the model construction was consistent with that used for the simulated cohorts (Fig. 1). We assessed the performance of each TSF-CNN model by calculating the mean and standard deviation of tenfold cross-validation AUC and test AUC. To determine whether the best performing model was statistically significantly better at classifying patients with T2D, we applied a two-sided Delong’s test to each model’s AUC versus the best performing model’s AUC^49^. The resulting *P* values were corrected for multiple testing using a false discovery rate (FDR) approach, and a FDR *P* < 0.05 was used as the threshold for statistical significance^50^.

### Reporting summary

Further information on research design is available in the Nature Research Reporting Summary linked to this article.

## Supplementary information

Reporting Summary

Supplementary Material

Data Set 1

## Data Availability

Simulated data cohorts are available in the following online repository online: 10.6084/m9.figshare.20205617.

## Code availability

All code is available at: https://github.com/rotroff-lab/Deep_learning_longitudinal_EHR_trajectories.
