# Supplementary material for: Identification of robust deep neural network models of longitudinal clinical measurements
Source: NPJ Digit Med. 2022 Jul 27;5:106. doi: 10.1038/s41746-022-00651-4 (PMC9329311; doi:10.1038/s41746-022-00651-4)
Supplement: Supplementary file 1 — Reporting Summary [file 41746_2022_651_MOESM1_ESM.pdf]

nature portfolio | reporting summary

nature portfolio

Corresponding author(s): Daniel Rotroff

Last updated by author(s): May 27, 2022

Reporting Summary

Nature Portfolio wishes to improve the reproducibility of the work that we publish. This form provides structure for consistency and transparency in reporting. For further information on Nature Portfolio policies, see our Editorial Policies and the Editorial Policy Checklist.

Statistics

For all statistical analyses, confirm that the following items are present in the figure legend, table legend, main text, or Methods section.

n/a	Confirmed	
☐	☒	The exact sample size (*n*) for each experimental group/condition, given as a discrete number and unit of measurement
☐	☒	A statement on whether measurements were taken from distinct samples or whether the same sample was measured repeatedly
☐	☒	The statistical test(s) used AND whether they are one- or two-sided<br>*Only common tests should be described solely by name; describe more complex techniques in the Methods section.*
☐	☒	A description of all covariates tested
☐	☒	A description of any assumptions or corrections, such as tests of normality and adjustment for multiple comparisons
☐	☒	A full description of the statistical parameters including central tendency (e.g. means) or other basic estimates (e.g. regression coefficient) AND variation (e.g. standard deviation) or associated estimates of uncertainty (e.g. confidence intervals)
☐	☒	For null hypothesis testing, the test statistic (e.g. *F*, *t*, *r*) with confidence intervals, effect sizes, degrees of freedom and *P* value noted<br>*Give P values as exact values whenever suitable.*
☒	☐	For Bayesian analysis, information on the choice of priors and Markov chain Monte Carlo settings
☒	☐	For hierarchical and complex designs, identification of the appropriate level for tests and full reporting of outcomes
☒	☐	Estimates of effect sizes (e.g. Cohen's *d*, Pearson's *r*), indicating how they were calculated

Our web collection on statistics for biologists contains articles on many of the points above.

Software and code

Policy information about availability of computer code

Data collection	No software was used.
Data analysis	All data analysis was performed using R version 4.1.1 and Python version 3.7. All code is available at: https://github.com/rotroff-lab/Deep_learning_longitudinal_EHR_trajectories, and this information is provided in the manuscript.

For manuscripts utilizing custom algorithms or software that are central to the research but not yet described in published literature, software must be made available to editors and reviewers. We strongly encourage code deposition in a community repository (e.g. GitHub). See the Nature Portfolio guidelines for submitting code & software for further information.

Data

Policy information about availability of data

All manuscripts must include a data availability statement. This statement should provide the following information, where applicable:

- Accession codes, unique identifiers, or web links for publicly available datasets
- A description of any restrictions on data availability
- For clinical datasets or third party data, please ensure that the statement adheres to our policy

Simulated data cohorts are available upon request.

Human research participants

Policy information about studies involving human research participants and Sex and Gender in Research.

Reporting on sex and gender	Self-reported sex, based on EHR records, is provided for real-world cohort. The analysis did not take sex into consideration as it was based on simulated data.
Population characteristics	The model that demonstrated the best performance across the simulated cohorts was evaluated in a real-world pediatric (ages 2-18 years) cohort to predict onsets of pediatric type 2 diabetes, prediabetes, and metabolic syndrome (Fig. 4A). The cohort consisted of 51,164 pediatric patients (54.9% male and 45.1% female based on self-report in the EHR) seen at the Cleveland Clinic between 1987 and 2020. Structured data, including height, weight, demographic information, encounter diagnosis international classification of disease (ICD) 9/10 codes, and medication records were extracted from the EHR. A detailed table of population characteristics are shown in Supplemental Table 1.
Recruitment	Patients were considered to have T2D based on the visit at which patients met the eMERGE criteria [Ref: 42]. Patients without a recorded encounter diagnosis or weight and height measurements were excluded. BMI was calculated from height and weight measurements from ages 2 to 12 years old or to the date of pediatric T2D diagnosis, whichever came first. Twelve years of age was selected as the max for BMI records because 12.5 years was the average age of a pediatric T2D diagnoses in this cohort; data prior to the age that most pediatric patients are diagnosed with T2D is desirable so that all models will be broadly applicable to most patients. Patients must also have had at least one encounter ICD code at or after the age of 18 to ensure they were still being seen by providers in the Cleveland Clinic health system. Additional selection criteria were implemented based on data availability and are described in the Data Processing and Quality Control section below.
Ethics oversight	This study was approved by Cleveland Clinic Institutional Review Board (IRB #20-1035)

Note that full information on the approval of the study protocol must also be provided in the manuscript.

Field-specific reporting

Please select the one below that is the best fit for your research. If you are not sure, read the appropriate sections before making your selection.

☒ Life sciences ☐ Behavioural & social sciences ☐ Ecological, evolutionary & environmental sciences

For a reference copy of the document with all sections, see nature.com/documents/nr-reporting-summary-flat.pdf

Life sciences study design

All studies must disclose on these points even when the disclosure is negative.

Sample size	The real-world cohort consisted of 51,164 pediatric patients. The cohort size was determined based on data availability and inclusion/ exclusion criteria.
Data exclusions	Patients without a recorded encounter diagnosis or weight and height measurements were excluded. BMI was calculated from height and weight measurements from ages 2 to 12 years old or to the date of pediatric T2D diagnosis, whichever came first. Twelve years of age was selected as the max for BMI records because 12.5 years was the average age of a pediatric T2D diagnoses in this cohort; data prior to the age that most pediatric patients are diagnosed with T2D is desirable so that all models will be broadly applicable to most patients. Patients must also have had at least one encounter ICD code at or after the age of 18 to ensure they were still being seen by providers in the Cleveland Clinic health system.
Replication	The cohort was randomly partitioned into a training/validation cohort (70%) and test cohort (30%). Model development was performed on the training/validation using cross-validation. The best model was then tested on the withheld test cohort.
Randomization	Patients were considered to have developed type 2 diabetes if they met the eMERGE criteria for type 2 diabetes. This criteria is based on a combination of ICD codes, medications, and lab results. Patients that did not meet this criteria and were considered to have not developed type 2 diabetes. Additional details and references are provided in the methods section of the manuscript.
Blinding	Blinding was not performed because this was a retrospective study. However, the test group was withheld from model training for an robust evaluation of model performance.

Reporting for specific materials, systems and methods

We require information from authors about some types of materials, experimental systems and methods used in many studies. Here, indicate whether each material, system or method listed is relevant to your study. If you are not sure if a list item applies to your research, read the appropriate section before selecting a response.

Materials & experimental systems

n/a	Involved in the study	
☒	☐	Antibodies
☒	☐	Eukaryotic cell lines
☒	☐	Palaeontology and archaeology
☒	☐	Animals and other organisms
☐	☒	Clinical data
☒	☐	Dual use research of concern

Methods

n/a	Involved in the study	
☒	☐	ChIP-seq
☒	☐	Flow cytometry
☒	☐	MRI-based neuroimaging

Clinical data

Policy information about clinical studies

All manuscripts should comply with the ICMJE guidelines for publication of clinical research and a completed CONSORT checklist must be included with all submissions.

Clinical trial registration: This was not a clinical trial

Study protocol: This was not a clinical trial

Data collection: The cohort consisted of 51,164 pediatric patients seen at the Cleveland Clinic between 1987 and 2020. Structured data, including height, weight, demographic information, encounter diagnosis international classification of disease (ICD) 9/10 codes, and medication records were extracted from the EHR. This process is described in the methods section of the manuscript.

Outcomes: Outcomes were defined as meeting the eMERGE criteria for type 2 diabetes. This is described above and in the methods text of the manuscript.
