# Supplementary material for: Identification of robust deep neural network models of longitudinal clinical measurements
Source: NPJ Digit Med. 2022 Jul 27;5:106. doi: 10.1038/s41746-022-00651-4 (PMC9329311; doi:10.1038/s41746-022-00651-4)
Supplement: Supplementary file 2 — Supplementary Material [file 41746_2022_651_MOESM2_ESM.docx]

Supplementary Information

Identification of Robust Optimal Deep Neural Network Models of Longitudinal Clinical Measurements

**Hamed Javidi^1,2^, Arshiya Mariam^1^, Gholamreza Khademi^1^, Emily Zabor^1^, Ran Zhao^1^, Tomas Radivoyevitch^1^, Daniel M. Rotroff^1,2,3,4,#^**

1. **Department of Quantitative Health Sciences, Lerner Research Institute, Cleveland Clinic, Cleveland, OH, USA**
2. **Department of Electrical Engineering and Computer Science, Cleveland State University, Cleveland, OH, USA**
3. **Endocrinology and Metabolism Institute, Cleveland Clinic, Cleveland, OH, USA**
4. **Cleveland Clinic Lerner College of Medicine, Case Western Reserve University, Cleveland, OH, USA**

**# Corresponding author**

Daniel M. Rotroff, PhD, MSPH

Department of Quantitative Health Sciences

Lerner Research Institute

Cleveland Clinic

9500 Euclid Avenue,

JJN3-01,

Cleveland, OH 44195, USA

Phone: 216-444-3399

Email: [rotrofd@ccf.org](mailto:rotrofd@ccf.org)

**Keywords:** Deep Neural Network, Pediatric records, Type-2 diabetes, Polycystic ovary syndrome, Longitudinal data, Electronic Health Record


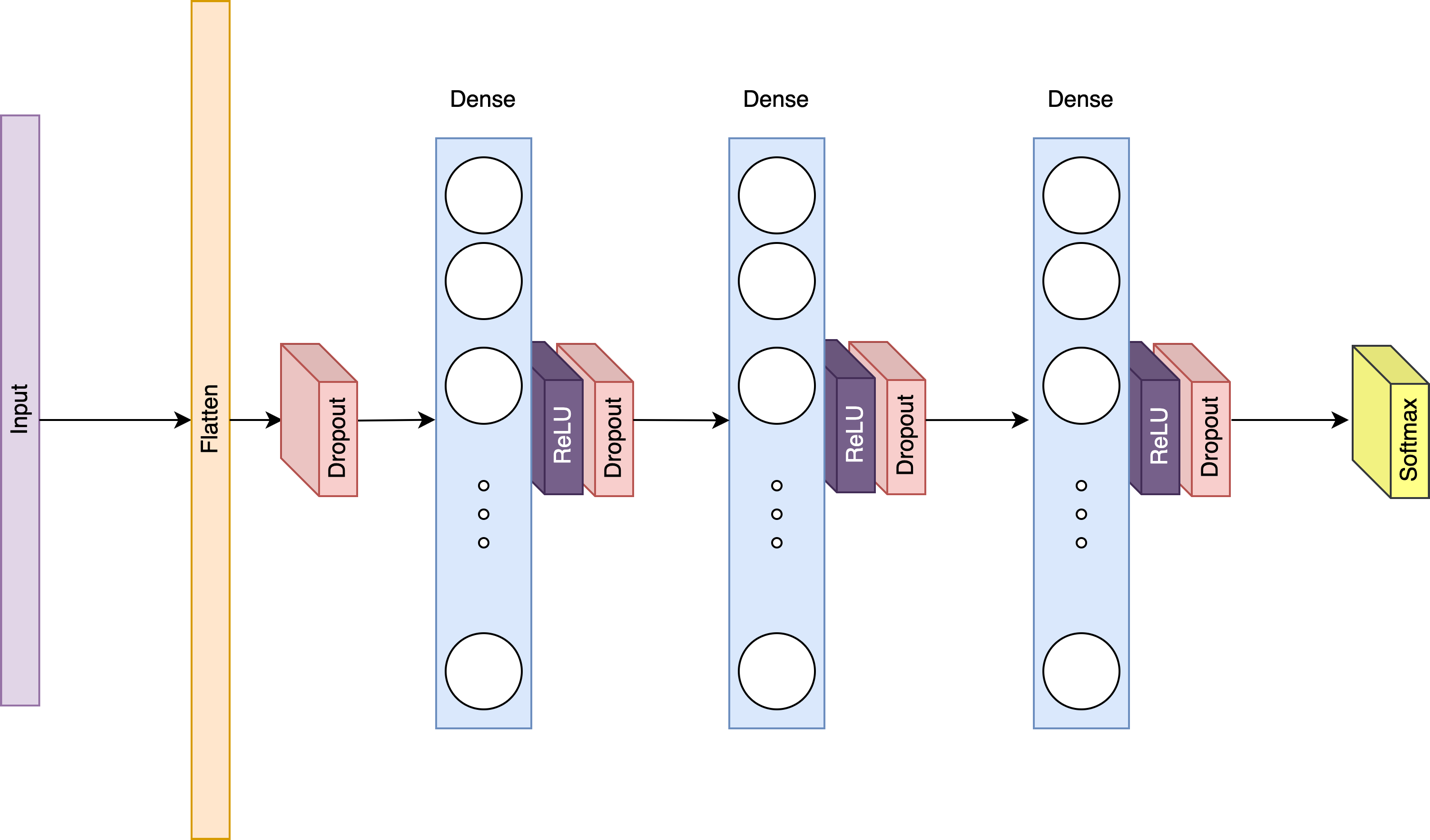


Supplementary Figure 1: Multi-Layer Perceptron (MLP) architecture


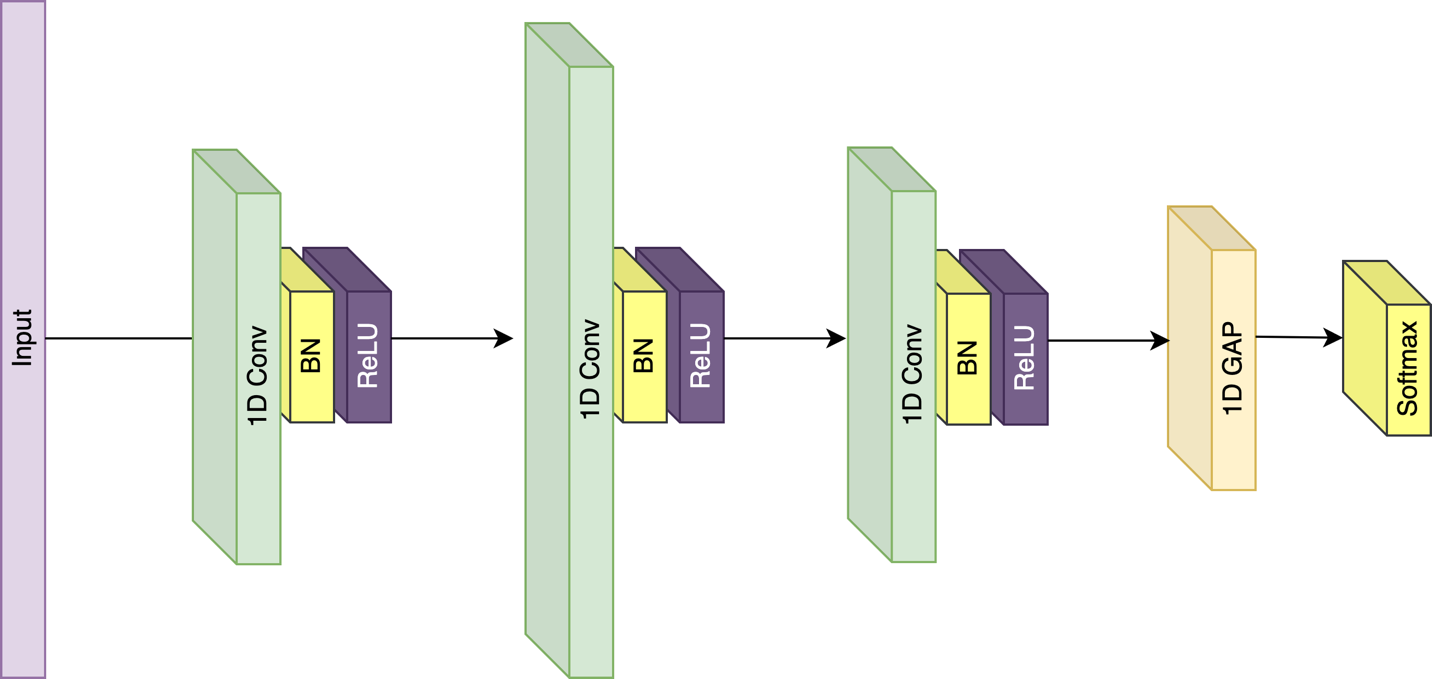


Supplementary Figure 2: Fully convolutional neural network (FCNN) architecture


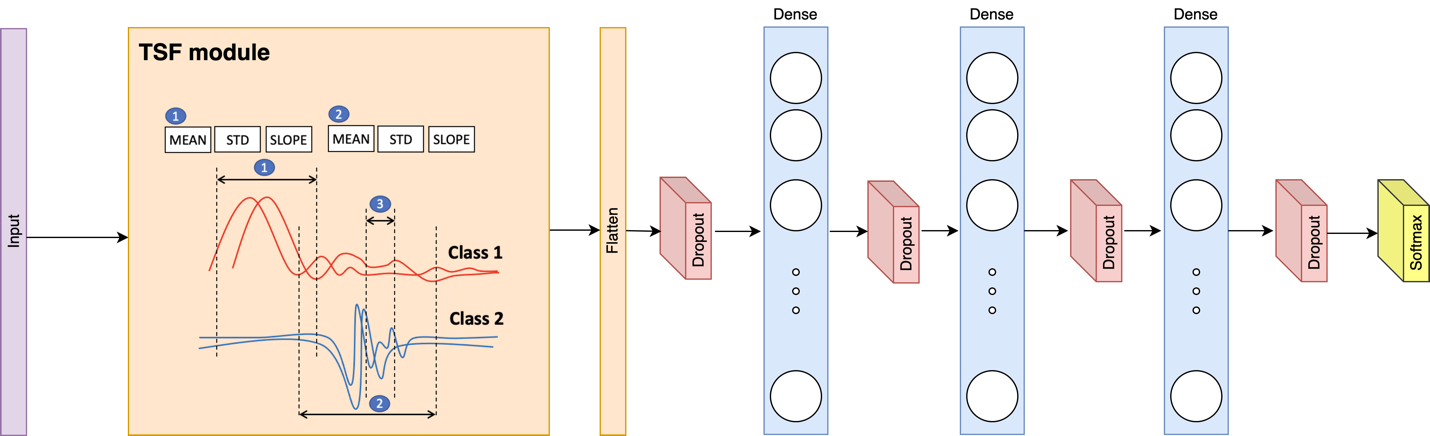


Supplementary Figure 3: Time series forest with multilayer perceptron (TSF-MLP) architecture


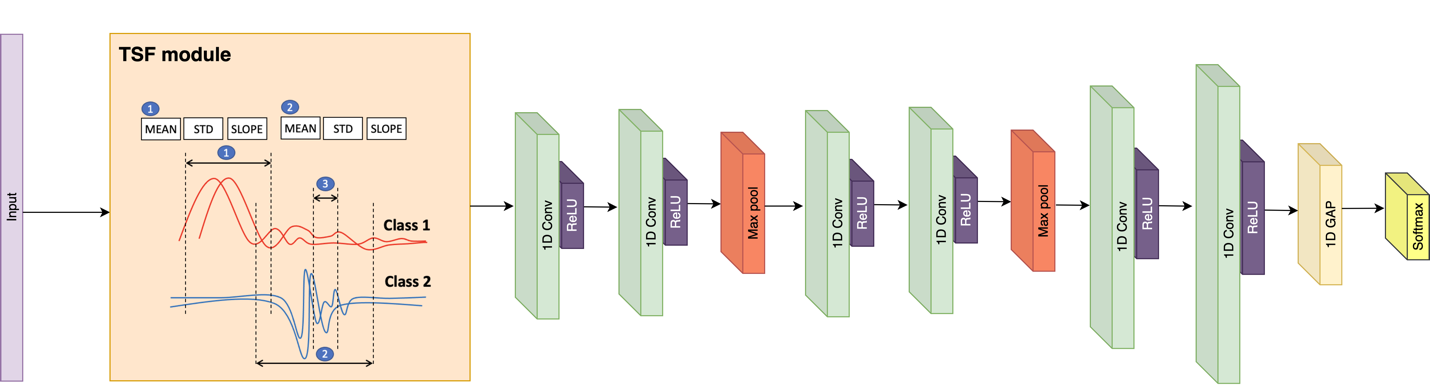


Supplementary Figure 4: Time series forest with convolutional neural network (TSF-CNN) architecture


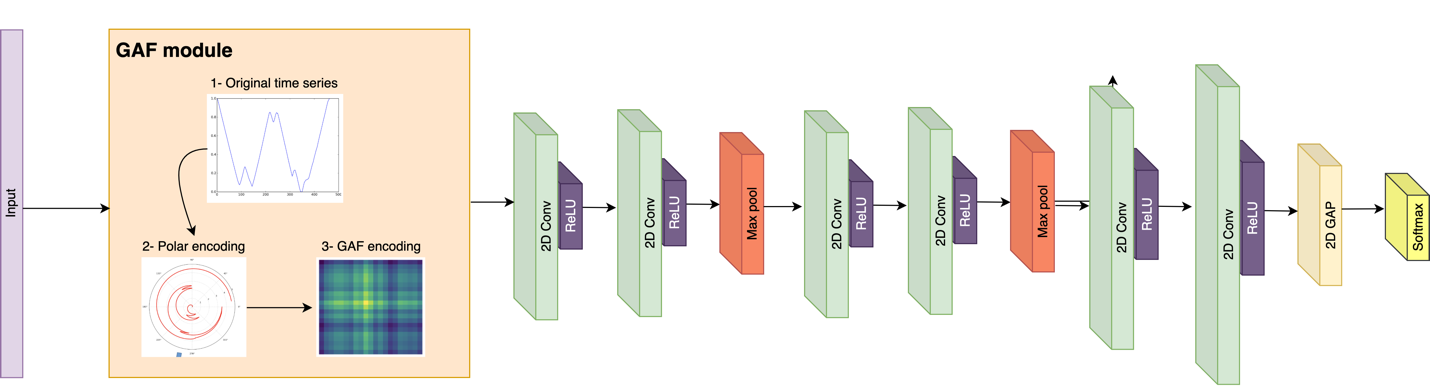


Supplementary Figure 5: Gramian angular field convolutional neural network (GAF-CNN) architecture


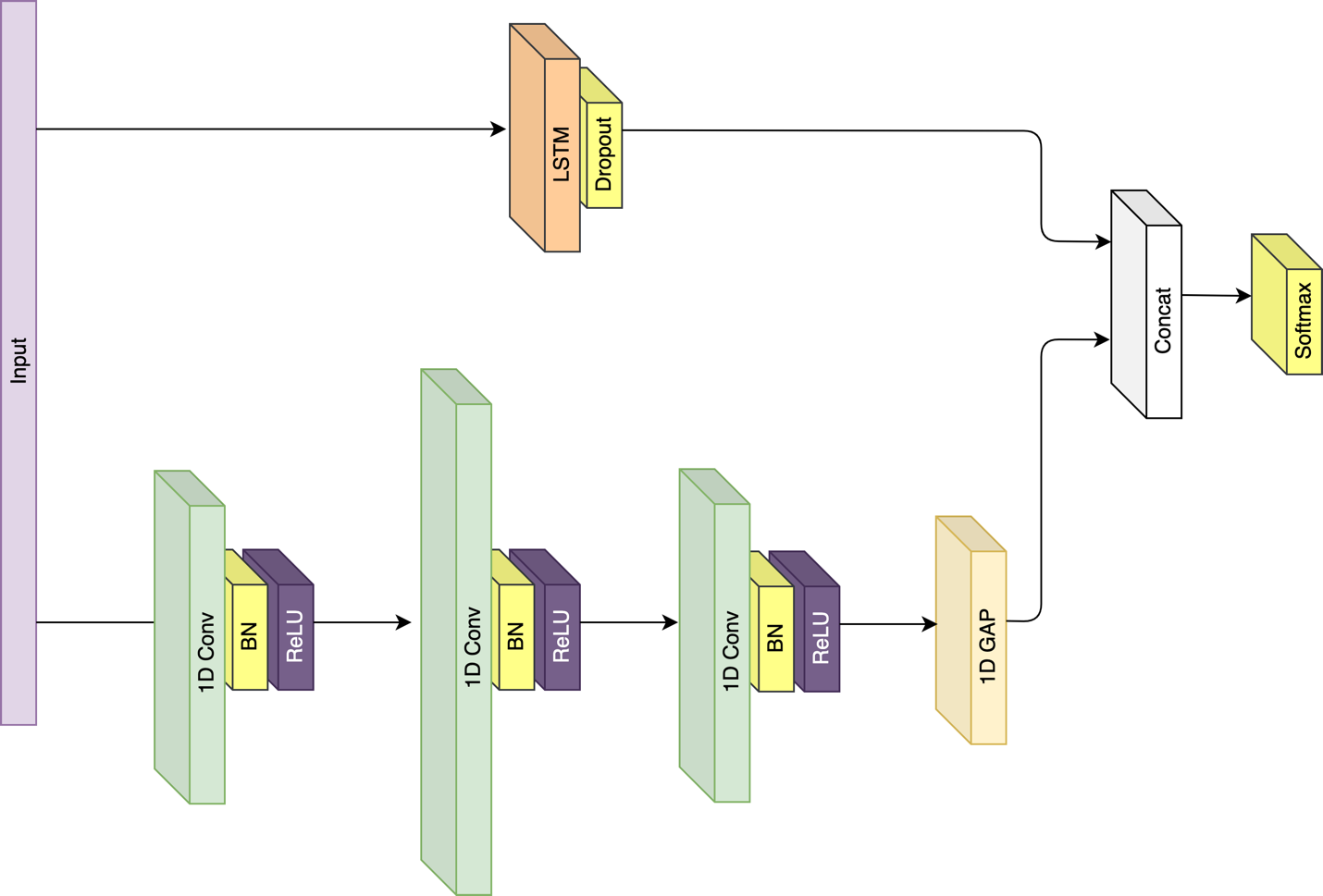


Supplementary Figure 6: RNN-FCN architecture


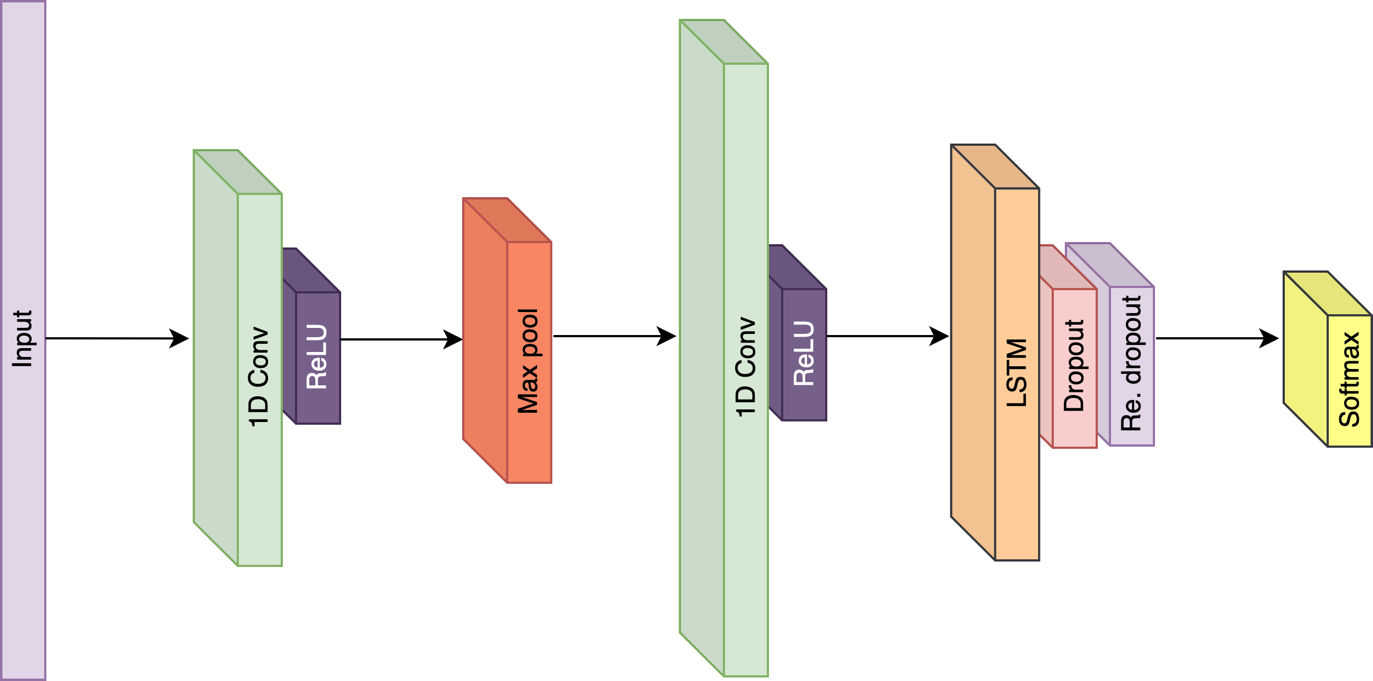


Supplementary Figure 7: Convolutional-Recurrent Neural Network architecture (CNN-RNN)


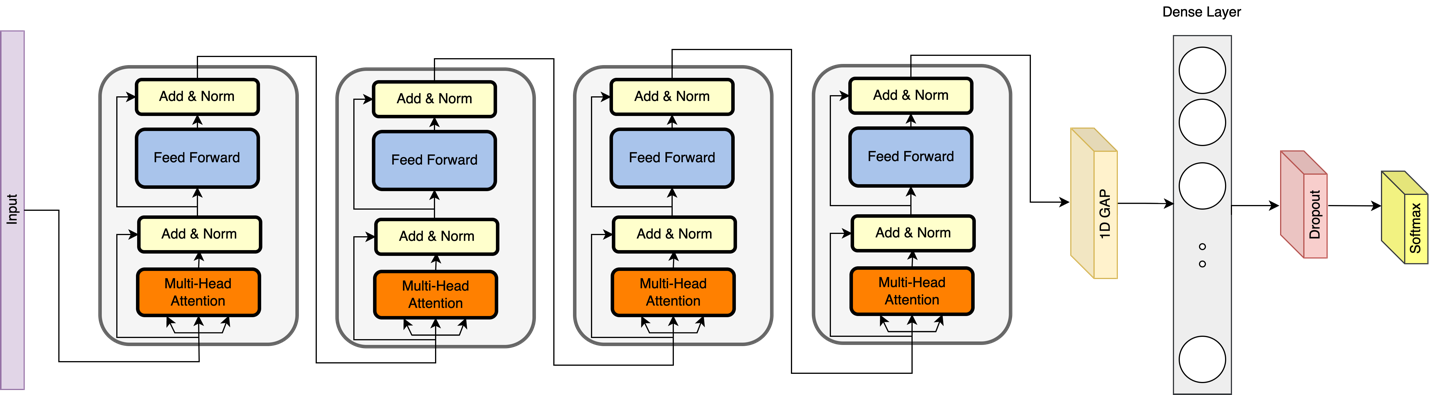


Supplementary Figure 8: Modified Transformer architecture for time series classification

Supplementary Table 1: Main cohort statistics

| Feature | Non-T2Ds | T2Ds | Total |
| --- | --- | --- | --- |
| Participants (n) | 32,720 | 2,336 | 35,056 |
| Age range (years) | 2-18 | 2-18 | 2-18 |
| Mean age at T2D diagnosis years (SD) | NA | 12.42 (3.42) | NA |
| Sex | | | |
| Males | 17971(55%) | 1036(44%) | 19007(54%) |
| Females | 14749 (45%) | 1300 (56%) | 16049 (46%) |
| Ethnicity | | | |
| Not Hispanic or Latino | 29890 (91%) | 2053 (88%) | 31943 (91%) |
| Hispanic or Latino | 1739 (5%) | 223 (10%) | 1962 (6%) |
| Unknown | 1091 (4%) | 60 (2%) | 1151 (3%) |
| Race | | | |
| Caucasian | 24031 (73%) | 1440 (62%) | 25471 (73%) |
| Black | 5336 (16%) | 536 (23%) | 5872 (17%) |
| Asian | 373 (1%) | 21 (1%) | 394 (1%) |
| Multiracial | 1538 (5%) | 192 (8%) | 1730 (5%) |
| Other | 1313 (4%) | 137 (6%) | 1450 (4%) |
| BMI Classification^1^ | | | |
| Underweight | 303 (1%) | 10 (1%) | 313 (1%) |
| Normal weight | 7268 (22%) | 284 (12%) | 7552 (22%) |
| Over-weight | 8373 (26%) | 361 (15%) | 8734 (25%) |
| Obese | 13539 (41%) | 948 (41%) | 14487 (41%) |
| Severe-obese | 3237 (10%) | 733 (31%) | 3970 (11%) |
| HbA1c | | | |
| Mean (SD) | 6.27 (1.92) | 7.01 (2.48) | 6.50 (2.10) |
| Health insurance | | | |
| Private health insurance | 17613 (54%) | 1073 (46%) | 18686 (53%) |
| Employee health insurance | 2962 (9%) | 236 (10%) | 3198 (9%) |
| Self-pay | 1518 (5%) | 81 (3%) | 1599 (5%) |
| Medicare/Medicaid | 8016 (24%) | 820 (35%) | 8836 (25%) |
| Unknown | 2737 (8%) | 126 (6%) | 2611 (8%) |
| Visits | | | |
| Mean patient visits per year | 1.31 | 1.74 | 1.34 |

^1^ ﻿BMI was converted to age and sex-specific BMI z-scores and percentiles using the CDC 2000 growth curves ^1^, then classified into underweight (<5th percentile), normal weight ( 5th, <85th), overweight ( 85th, <95th), obesity ( 95th to <20% higher than the 95th percentile), and severe obesity ( 20% higher than the 95th percentile) ^2^ .

Supplementary Table 2: Design parameters for the deep learning approaches

| Methods |  | | |  | | | | | | |  |
| --- | --- | --- | --- | --- | --- | --- | --- | --- | --- | --- | --- |
|  | #Layer | | #Conv | | #LSTM | Normalize | Pooling | Feature | Active | Regularize | Loss function |
| MLP | | 4 | 0 | | 0 | None | None | FC | ReLU | Dropout | Binary cross entropy |
| FCN | | 5 | 3 | | 0 | Batch | None | GAP | ReLU | None | Binary cross entropy |
| TSF-MLP | | 4 | 0 | | 0 | None | None | FC | ReLU | Dropout | Binary cross entropy |
| TSF-CNN | | 7 | 6 | | 0 | None | Max | GAP | ReLU | None | Binary cross entropy |
| GAF-CNN | | 6 | 5 | | 0 | None | Max | GAP | ReLU | None | Binary cross entropy |
| ResNet | | 11 | 9 | | 0 | Batch | None | GAP | ReLU | None | Binary cross entropy |
| RNN-FCN | | 7 | 3 | | 1 | Batch | None | GAP | ReLU | Dropout | Binary cross entropy |
| CNN-RNN | | 4 | 2 | | 1 | None | Max | None | None | Dropout | Binary cross entropy |
| Transformer | | 42 | 0 | | 0 | None | None | None | ReLU | Dropout | Binary cross entropy |

Supplementary Table 3: Hyperparameter tuning variables and ranges

| Category | Name | Type | Range | Optimizer method |
| --- | --- | --- | --- | --- |
| Shared | Batch size | Int | [64, 128, 256, 512] | Hyperband |
|  | Drop out | Float | [0.1-0.8] | Hyperband |
| Convolution | Kernel size | Int | [32, 64, 128] | Hyperband |
|  | Kernel width | Int | [3, 5, 8] | Hyperband |
| LSTM | # block | Int | [4, 8, 16, 32] | Hyperband |
|  | Width | Int | [3, 5, 8] | Hyperband |
|  | Recurrent drop out | Float | [0.1-0.8] | Hyperband |
| Attention | # Head | Int | [1, 2, 4, 8] | Hyperband |
|  | Head size | Int | [1, 2, 4, 8] | Hyperband |
|  | Feed forward | Int | [1, 2, 4, 8] | Hyperband |


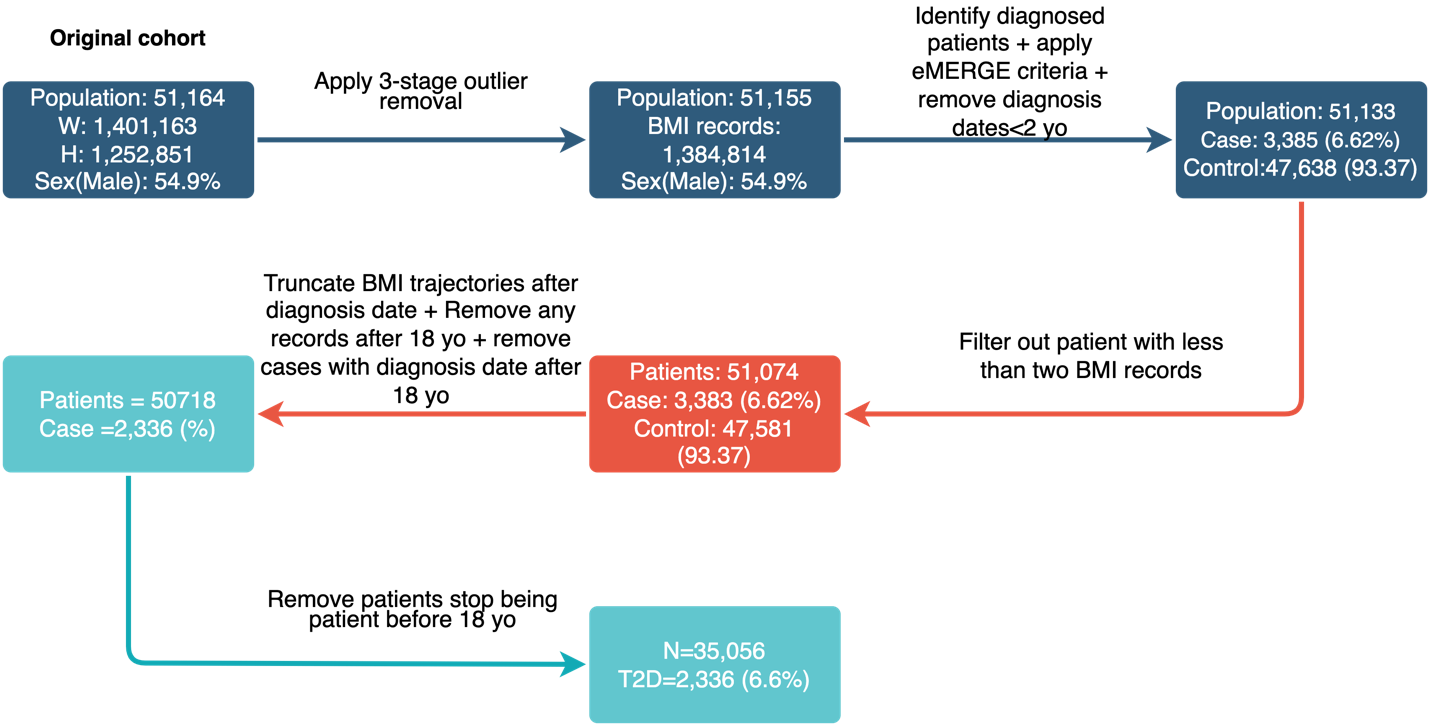


Supplementary Figure 9: Detailed STROBE diagram of data processing steps. Step1: Our outlier detection method eliminated 9 patients. Step 2: The eMERGE criteria^3^ was applied to patients with any relevant diagnosis code to define those with type 2 diabetes (T2D); those with T2D before 2 years of age were removed. Step 3: Patients with <2 BMI records were removed. Step 4: Patient BMI trajectories were truncated to those before the diagnosis date. Anyone diagnosed after the age of 18 years was removed. Step 5: Patients lacking follow up through 18 years of age were excluded.


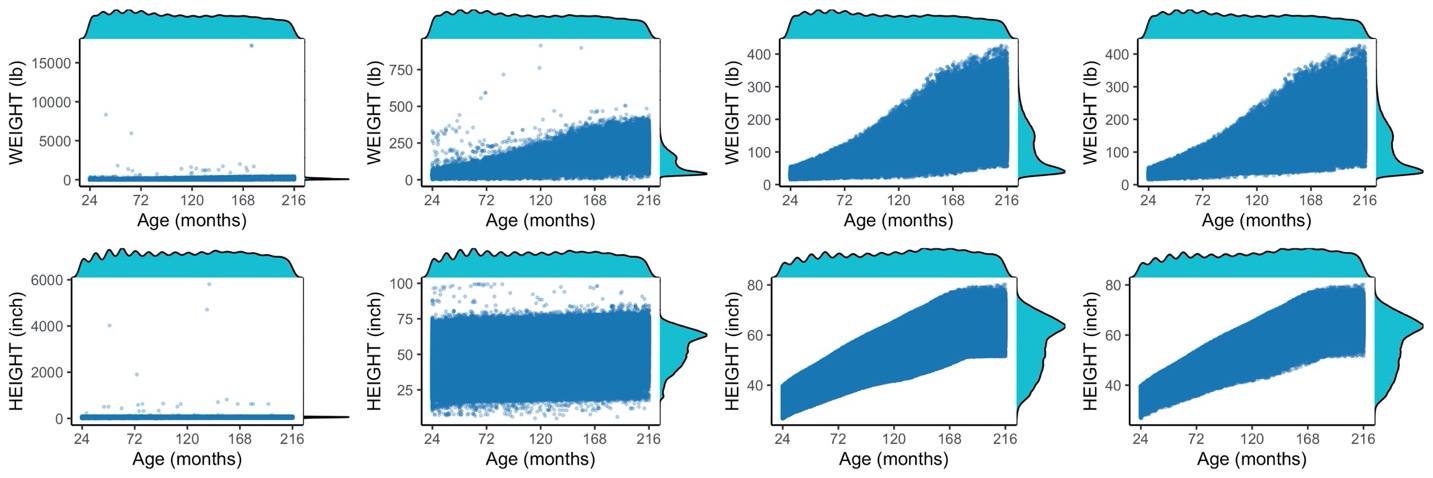


Supplementary Figure 10: 3-stage outlier removal. 1^st^ column: before removal; 2^nd^ column: After the 1^st^ stage of outlier removal; 3^rd^ column: After the 2^nd^ stage; 4^th^ column: final weights and heights

Supplementary Figure 11. Effect of distribution overlap on the model’s mean area under the receiver operating characteristic curve (AUC) when there is no missing data and the data is without irregularities.

Supplementary Figure 12: Effect of irregularity on model predictive accuracies (A) Mean area under the receiver operating characteristic curve (AUC) of the test set for the simulated magnitude cohort across all models. (B) Mean Test AUC for the simulated shape cohort across all models. The x-axis represents different effect sizes, and the y-axis represents different measures of dispersion, categorized by irregularity. The colors are scaled by the mean test AUC for each model.

Supplementary Figure 13: Effect of class imbalanced ratio on models’ performance


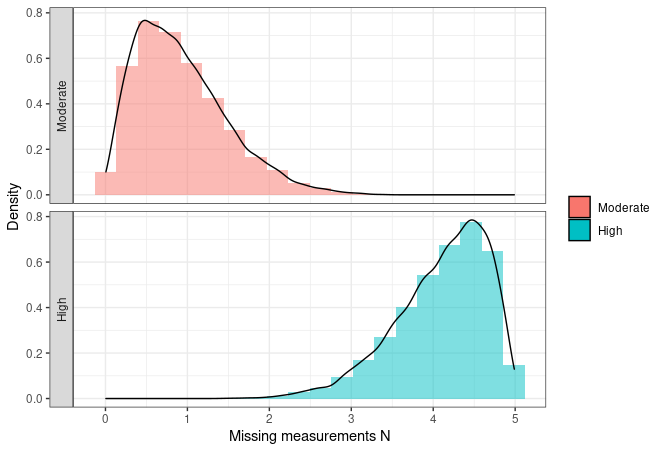


Supplementary Figure 14: Beta distribution used to sample how many missing data points would be applied to each patient’s trajectory in those classified as having moderate or high irregularity. For the moderate irregularity beta distribution, the parameters used were alpha=2 and beta=9. For the high irregularity beta distribution, the parameters used were alpha=9 and beta=2.

Supplementary Figure 15: **Model predictive accuracies on simulated data sets by effect size, dispersion, and missingness only for BMI cohort.** Cohorts include all class imbalance ratios, with no irregularity present (A) Mean area under the receiver operating characteristic curve (AUC) of the test set for the simulated magnitude cohort across BMI cohort. (B) Mean Test AUC for the simulated shape cohort across BMI cohorts. The x-axis represents different effect sizes, and the y-axis represents different measures of dispersion, categorized by % data missingness. The colors are scaled by the mean AUC for each model.

Supplementary Figure 16: **Model predictive accuracies on simulated data sets by effect size, dispersion, and missingness only for SBP cohort.** Cohorts include all class imbalance ratios, with no irregularity present (A) Mean area under the receiver operating characteristic curve (AUC) of the test set for the simulated magnitude cohort across SBP cohort. (B) Mean Test AUC for the simulated shape cohort across SBP cohorts. The x-axis represents different effect sizes, and the y-axis represents different measures of dispersion, categorized by % data missingness. The colors are scaled by the mean AUC for each model.

Supplementary Figure 17: **Model predictive accuracies on simulated data sets by effect size, dispersion, and missingness only for glucose cohort.** Cohorts include all class imbalance ratios, with no irregularity present (A) Mean area under the receiver operating characteristic curve (AUC) of the test set for the simulated magnitude cohort across glucose cohort. (B) Mean Test AUC for the simulated shape cohort across glucose cohorts. The x-axis represents different effect sizes, and the y-axis represents different measures of dispersion, categorized by % data missingness. The colors are scaled by the mean AUC for each model.

Supplementary Table 4: Type-2 Diabetes cohort statistics

| Ages (years) | Cohort size  (N) | T2D status %  Positive | Sex %  Female | Race %  White | Val AUC  Mean (sd) | Test AUC  Mean (sd) |
| --- | --- | --- | --- | --- | --- | --- |
| 2 to 4 | 5,223 | 4.1 | 42.7 | 81.2 | 0.61 (0.07) | 0.50 (0.01) |
| 2 to 5 | 5,210 | 3.9 | 42.7 | 81.2 | 0.61 (0.09) | 0.58 (0.03) |
| 2 to 6 | 5,164 | 3.6 | 42.7 | 81.3 | 0.53 (0.14) | 0.52 (0.01) |
| 2 to 7 | 4,592 | 3.7 | 42.6 | 81.5 | 0.55 (0.08) | 0.63 (0.01) |
| 2 to 8 | 4,091 | 3.7 | 42.7 | 82.2 | 0.61 (0.11) | 0.60 (0.04) |
| 2 to 9 | 3,668 | 3.7 | 42.5 | 82.2 | 0.59 (0.18) | 0.56 (0.03) |
| 2 to 10 | 3,192 | 3.9 | 42.7 | 82.8 | 0.68 (0.12) | 0.64 (0.01) |
| 2 to 11 | 2,803 | 3.8 | 42.6 | 82.8 | 0.67 (0.08) | 0.69 (0.00) |
| 2 to 12 | 2,500 | 3.3 | 42.6 | 83.1 | 0.71 (0.19) | 0.63 (0.01) |
| 3 to 5 | 10,193 | 4.8 | 43.7 | 76.4 | 0.63 (0.07) | 0.58 (0.01) |
| 3 to 6 | 10,109 | 4.6 | 43.7 | 76.5 | 0.63 (0.09) | 0.66 (0.01) |
| 3 to 7 | 9,244 | 4.7 | 43.6 | 76.4 | 0.64 (0.07) | 0.65 (0.01) |
| 3 to 8 | 8,374 | 4.8 | 43.7 | 77 | 0.65 (0.06) | 0.69 (0.01) |
| 3 to 9 | 7,602 | 4.8 | 43.4 | 77.2 | 0.69 (0.10) | 0.66 (0.01) |
| 3 to 10 | 6,807 | 4.9 | 43.3 | 77.7 | 0.67 (0.09) | 0.62 (0.01) |
| 3 to 11 | 6,076 | 4.6 | 43.5 | 78.3 | 0.65 (0.12) | 0.69 (0.01) |
| 3 to 12 | 5,450 | 4.2 | 43.5 | 78.9 | 0.74 (0.08) | 0.72 (0.01) |
| 4 to 6 | 12,827 | 5.2 | 43.9 | 75.6 | 0.65 (0.06) | 0.61 (0.00) |
| 4 to 7 | 11,940 | 5.3 | 43.8 | 75.5 | 0.67 (0.06) | 0.67 (0.01) |
| 4 to 8 | 10,984 | 5.3 | 43.9 | 75.9 | 0.66 (0.05) | 0.68 (0.00) |
| 4 to 9 | 10,074 | 5.4 | 43.6 | 76.1 | 0.69 (0.04) | 0.69 (0.01) |
| 4 to 10 | 9,152 | 5.4 | 43.5 | 76.6 | 0.64 (0.02) | 0.64 (0.00) |
| 4 to 11 | 8,294 | 5.1 | 43.7 | 77 | 0.67 (0.09) | 0.69 (0.00) |
| 4 to 12 | 7,501 | 4.6 | 43.8 | 77.6 | 0.71 (0.06) | 0.68 (0.00) |
| 5 to 7 | 14,357 | 5.6 | 44.2 | 75.2 | 0.66 (0.06) | 0.64 (0.00) |
| 5 to 8 | 13,388 | 5.7 | 44.4 | 75.5 | 0.67 (0.03) | 0.65 (0.01) |
| 5 to 9 | 12,413 | 5.7 | 44.1 | 75.7 | 0.68 (0.04) | 0.64 (0.00) |
| 5 to 10 | 11,379 | 5.7 | 44.1 | 76.1 | 0.68 (0.03) | 0.68 (0.01) |
| 5 to 11 | 10,411 | 5.4 | 44.2 | 76.4 | 0.65 (0.07) | 0.62 (0.00) |
| 5 to 12 | 9,520 | 4.9 | 44.4 | 77 | 0.67 (0.05) | 0.71 (0.01) |
| 6 to 8 | 15,319 | 6 | 44.5 | 75.2 | 0.67 (0.04) | 0.66 (0.00) |
| 6 to 9 | 14,324 | 6 | 44.3 | 75.4 | 0.69 (0.04) | 0.65 (0.00) |
| 6 to 10 | 13,252 | 5.9 | 44.3 | 75.8 | 0.67 (0.06) | 0.65 (0.01) |
| 6 to 11 | 12,204 | 5.7 | 44.3 | 76.1 | 0.68 (0.06) | 0.68 (0.00) |
| 6 to 12 | 11,210 | 5.2 | 44.4 | 76.6 | 0.67 (0.05) | 0.64 (0.00) |
| 7 to 9 | 16,039 | 6.3 | 44.2 | 74.9 | 0.68 (0.04) | 0.64 (0.00) |
| 7 to 10 | 14,951 | 6.2 | 44.2 | 75.2 | 0.66 (0.03) | 0.68 (0.00) |
| 7 to 11 | 13,848 | 6 | 44.2 | 75.5 | 0.67 (0.02) | 0.65 (0.00) |
| 7 to 12 | 12,774 | 5.6 | 44.3 | 75.9 | 0.68 (0.05) | 0.64 (0.01) |
| 8 to 10 | 16,865 | 6.5 | 44.2 | 74.7 | 0.68 (0.02) | 0.66 (0.00) |
| 8 to 11 | 15,741 | 6.2 | 44.2 | 75 | 0.66 (0.03) | 0.66 (0.00) |
| 8 to 12 | 14,613 | 5.8 | 44.3 | 75.3 | 0.66 (0.04) | 0.66 (0.00) |
| 9 to 11 | 17,802 | 6.3 | 44.5 | 74.6 | 0.66 (0.04) | 0.67 (0.01) |
| 9 to 12 | 16,658 | 5.8 | 44.7 | 74.9 | 0.68 (0.03) | 0.67 (0.00) |
| 10 to 12 | 18,847 | 5.9 | 44.7 | 74.5 | 0.69 (0.04) | 0.68 (0.01) |

Supplementary Figure 18: Regression analysis of AUC versus (a) number of cases (T2Ds) for all cohorts (b) number of controls (non-T2Ds) (c) cohort size (T2Ds+non-T2Ds) (d) age at the end of trajectory (e) age at the start of trajectory (f) length of trajectory

**Supplementary File 1.** File containing AUCs across different simulated conditions for each model.

**Supplementary References:**

1. Kuczmarski RJ, Ogden CL, Guo SS, Grummer-Strawn LM, Flegal KM, Mei Z, et al. 2000 CDC Growth Charts for the United States: Methods and Development. *Vital Heal. Stat* **11**, (2002).

2. Skinner, A. C., Ravanbakht, S. N., Skelton, J. A., Perrin, E. M. & Armstrong, S. C. Prevalence of Obesity and Severe Obesity in US Children, 1999–2016. *Pediatrics* **141**, 1999–2016 (2018).

3. Kho, A. N. *et al.* Use of diverse electronic medical record systems to identify genetic risk for type 2 diabetes within a genome-wide association study. *J. Am. Med. Informatics Assoc.* **19**, 212–218 (2012).
